# Population genetic structure and ecological differentiation in the bryozoan genus *Reteporella* across the Azores Archipelago (central North Atlantic)

**DOI:** 10.1016/j.heliyon.2024.e38765

**Published:** 2024-10-01

**Authors:** Lara Baptista, Manuel Curto, Andrea Waeschenbach, Björn Berning, António M. Santos, Sérgio P. Ávila, Harald Meimberg

**Affiliations:** a CIBIO-InBIO, Universidade dos Açores, Departamento de Biologia, Rua Mãe de Deus 13A, 9501-801, Ponta Delgada, São Miguel, Portugal; b BIOPOLIS Program in Genomics, Biodiversity and Land Planning, CIBIO, Campus de Vairão, Vairão, 4485-661, Portugal; c Institute for Integrative Nature Conservation Research, Department of Integrative Biology and Biodiversity Research, BOKU University, Gregor-Mendel-Straße 33, 1180, Wien, Austria; d CIBIO, Centro de Investigação em Biodiversidade e Recursos Genéticos, InBIO Laboratório Associado, Campus de Vairão, Universidade do Porto, Rua Padre Armando Quintas, no. 7, 4485-661, Vairão, Portugal; e Science, Natural History Museum, Cromwell Road, London, SW7 5BD, United Kingdom; f Faculdade de Ciências da Universidade do Porto, Rua do Campo Alegre 1021/1055, 4169-007, Porto, Portugal; g UNESCO Chair – Land Within Sea: Biodiversity & Sustainability in Atlantic Islands, Universidade dos Açores, 9501-801, Ponta Delgada, Portugal; h Departamento de Biologia, Faculdade de Ciências e Tecnologia, Universidade dos Açores, 9501-801, Ponta Delgada, Açores, Portugal

**Keywords:** Cryptic species, Incipient speciation, Bathymetry, SSR-GBAS, Azores Archipelago

## Abstract

The processes shaping population dynamics of benthic marine invertebrates with non-planktotrophic larvae are still poorly understood but have seen a renewed interest in applying integrative taxonomic approaches. We used mitochondrial and microsatellite (SSR-GBAS) data to estimate connectivity across islands and seamounts in the central North Atlantic Azores Archipelago in five species of the bryozoan genus *Reteporella* Busk, 1884. Discordant patterns were inferred between datasets, which might be due to methodological constraints related to the application of multilocus approaches based on amplification to multiple species or due to interspecific introgression in deep waters. A divergent cryptic ecotype of *Reteporella atlantica* (Busk, 1884) was found in shallow waters, likely resulting from ecologically-driven incipient speciation, posing new questions regarding the role of bathymetrical zonation as a promoter of differentiation. The occurrence of ecologically-driven differentiation and potential interspecific introgression in other bryozoans should be considered, both with potentially important evolutionary and biogeographical consequences. The discovery of incipient species, prompted by ecological factors, calls for the need to consider marine invertebrates when developing conservation strategies in oceanic insular ecosystems.

## Highlights

• Genetic connectivity across the Azores explored in 5 *Reteporella* species.
• Potential cryptic speciation of *Reteporella atlantica* from shallow waters.
• Discordant population patterns due to methodological constraints.
• Interspecific introgression might occur in deep waters.

## Graphical abstract

**Figure:**
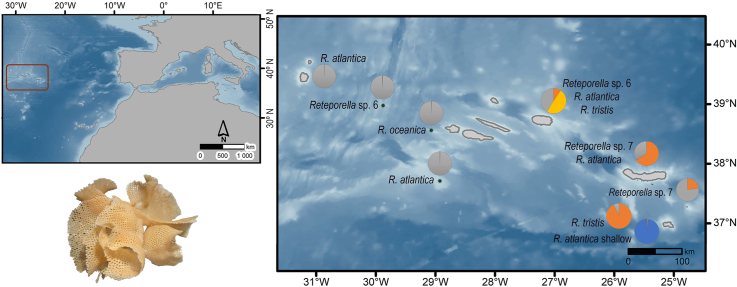

## 1 Introduction

Bryozoans are among the most overlooked invertebrate phyla and many aspects of their ecology, biology, and evolution remain unknown. Whilst the few existing phylogenetic studies were mostly conducted at higher systematic levels in an attempt to understand better the evolution of the phylum [[1], [2], [3]], most population genetic studies in bryozoans are focused on species’ complexes or invasive species such as *Celleporella hyalina* (Linnaeus, 1767) and *Amathia verticillata* (delle Chiaje, 1822) [4,5], or on genera containing such species, such as *Bugula* Oken, 1815 and *Watersipora* Neviani, 1896 [[6], [7], [8]]. Although the bryozoan diversity in some areas of the globe seems to be well documented [[9], [10], [11], [12], [13], [14]], modern taxonomic revisions of allegedly well-known genera or species usually result in a doubling of species numbers, even in well-studied regions such as the Mediterranean Sea [[15], [16], [17]]. In less well-studied ecosystems, such as the seamounts and islands of the oceanic Azores Archipelago in the central North Atlantic, limited sampling usually impedes a thorough diversity assessment, and taxonomic revisions of the few historical records using modern imaging and molecular techniques are scarce or lacking altogether [18,19]. In an attempt to tackle these problems, the Azorean species of the genus *Reteporella* Busk, 1884*,* the most widespread of the 23 currently recognized genera within the family Phidoloporidae, were recently studied following an integrative taxonomic approach, unravelling a distinctly greater diversity than previously documented and clarifying the phylogenetic relationships among the different species in the archipelago [20].

The Azores Archipelago is considered a hotspot of biodiversity in the North Atlantic, thriving due to its isolation, geological evolution, complex geomorphology, temperate climate, varied environments, and oceanographical setting [21,22]. Nine islands and over 400 seamounts [23], divided into three groups (eastern, central, and western) comprise this archipelago, which distances 820 km to the closest archipelago (Madeira) and over 1300 km to the continental shelves of America and Europe. Sea-surface circulation in the North Atlantic is complex, generally flowing eastwards in the Azores under the influence of the Gulf Stream [24,25].

The uniqueness of the Azores ecoregion (*sensu* [26]) is expressed in the increasing number of endemic species reported, as well as taxa previously regarded as widely distributed that are potential cryptic or sibling species restricted to the archipelago (e.g., microgastropods of the family Rissoidae [27,28] or deep-water corals of several genera [29]). Azorean bryozoans are particularly rich in endemic taxa, with most of the genera containing endemic species, while seven monotypic genera (e.g., *Nimbella* Jullien, 1903, *Pulpeirina* Reverter-Gil & Souto, 2015, or *Harmelinius* Rosso, 2018) are endemic to the archipelago. Intra-archipelagic gene flow and connectivity of marine species with non-planktotrophic larvae in the Azores have seen a renewed interest as a result of the development of new molecular techniques and integrative approaches. At least for intertidal species, several factors such as habitat type, sea-surface circulation regimes, and dispersal ability, seem to act concomitantly to shape population [27]. However, the full range of processes influencing the population dynamics remains poorly studied for benthic inhabitants of remote islands.

**Fig. 1:**
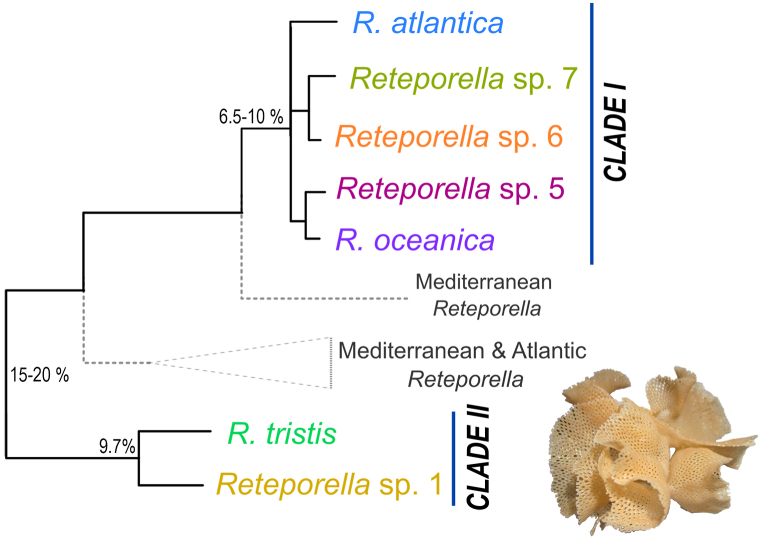
Dendrogram representing the phylogenetic relationships of *Reteporella* species in the Azores. Two clades are distinguished (I and II), diverging over 15 % in the COI marker; mitochondrial divergence ranges from 6.5 to 10 % within Clade I. Grey dotted branches represent Mediterranean and non-Azorean Atlantic *Reteporella* species. *Reteporella* colony is merely illustrative. Adapted from Baptista et al. [20]; accession numbers, outgroups and further inferences on the relationships between *Reteporella* species can be consulted therein.

In light of a recent review of their insular diversity [20], *Reteporella* species become an interesting proxy for exploring the genetic structure and population dynamics of benthic marine invertebrates in the Azores Archipelago. Occurring from 10 to 820 m depths on the islands and seamounts, *Reteporella* species occupy wide bathymetrical and geographical ranges [20], producing relatively large, erect colonies with a reticulate branching pattern. Baptista et al. [20], based on morphological and molecular data, retrieved two clades of Azorean *Reteporella* species (Fig. 1): Clade I comprises five closely related species – *Reteporella atlantica* (Busk, 1884)*, Reteporella oceanica* (Jullien in Jullien & Calvet, 1903), and *Reteporella* spp. 5–7 – whilst Clade II comprises *Reteporella tristis* (Jullien in Jullien & Calvet, 1903) and *Reteporella* sp. 1.

Species of the genus *Reteporella* possess non-planktotrophic larval development, thus lack a feeding stage and are relatively short-lived. Accordingly, their larvae are thought to have restricted dispersal abilities, thus genetic structure should be pronounced across the archipelago, reflecting geographical distance [[30], [31], [32]]. Aiming to estimate connectivity and explore the factors shaping population dynamics across Azorean islands and seamounts, the population genetics of five *Reteporella* species – *R. atlantica*, *R. tristis*, *R. oceanica,* and *Reteporella* spp. 6 and 7 (*sensu* [20]) – was studied. *Reteporella* sp. 5 was not included as only one locality yielded a single specimen. Microsatellite genotyping (by SSR-GBAS, see Material and methods) and COI haplotyping were used to account for reticulate nuclear and mitochondrial variation patterns, respectively. The congruence of genetic structure to species boundaries, sampling locality, and habitat was evaluated.

## 2 Material and methods

### 2.1 Sampling and DNA extraction

**Fig. 2:**
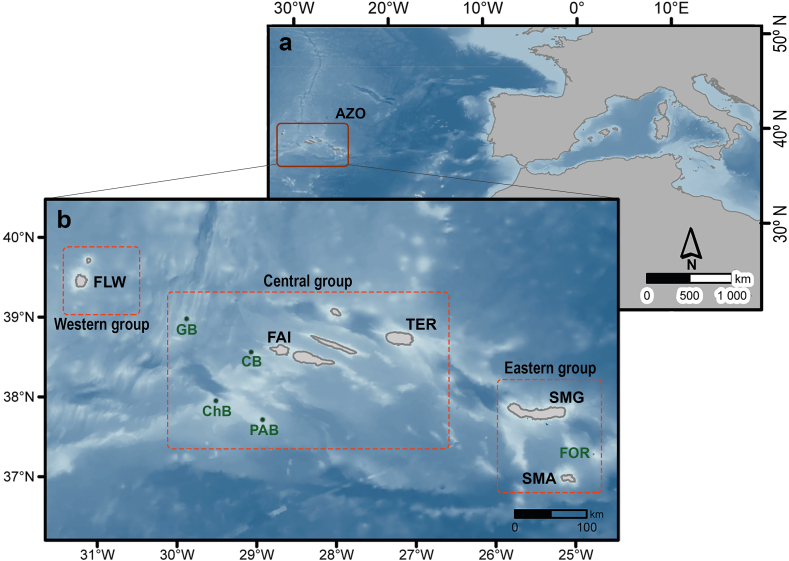
Geographical setting of the Azores Archipelago and localities sampled. Abbreviations are depicted in black for islands (SMA – Santa Maria, SMG – São Miguel, TER – Terceira, FAI – Faial, FLW – Flores) and green for seamounts (FOR – Formigas, PAB – Princess Alice Bank, ChB – Chino Bank, CB – Condor Bank, GB – Gigante Bank). Island/seamount groups in the Azores (eastern, central, and western groups) are delimited by dashed orange boxes. Coastline retrieved from the Portuguese Hydrographic Institute (https://www.hidrografico.pt/op/33) and bathymetry derived from GEBCO 2020 (https://www.gebco.net/data_and_products/gridded_bathymetry_data), with darker shades indicating water depths down to 4500 m. Adapted from Baptista et al. [20]. (For interpretation of the references to colour in this figure legend, the reader is referred to the Web version of this article.)

*Reteporella* specimens from ten locations around the Azores Archipelago (Fig. 2) were sub-sampled from material deposited at biological collections (DBUA – Department of Biology of the University of the Azores, and DOP – Department of Oceanography and Fisheries of the University of the Azores) or from material collected during the scientific cruise M150 “Controls in benthic and pelagic BIODIversity of the AZores BIODIAZ” with the German RV *Meteor* in 2018 [33] (see Table S1 for further details). All collected samples were preserved in ethanol >96 % and stored at a maximum temperature of 4 °C. Total genomic DNA was extracted with the column-based commercial kit PureLink® Genomic DNA (Invitrogen^TM^), after maceration of clean colony fragments with metallic pestles. The quality and integrity of the DNA extracted were evaluated with agarose gel electrophoresis (0.8 %).

### 2.2 Molecular work for SSR-GBAS genotyping

Genetic variation of *Reteporella* was assessed by short sequence repeat genotyping by amplicon sequencing (SSR-GBAS), which has been shown to be highly effective in assessing genetic structure in a variety of non-model organisms, including marine invertebrates [27,34,35].

*De novo* SSR-GBAS markers were developed from shot-gun sequencing data from two low-coverage Illumina MiSeq runs performed on *Reteporella atlantica* from Flores (reference library BRY16) and *Reteporella* sp. 6 from Terceira (reference library BRY141). Raw reads were deposited in the GenBank short read archive (BioProject number PRJNA1130121). Library preparation with the Nextera XT DNA Library Preparation Kit (Illumina, Inc.) and paired-end sequencing (2 x 300 bp reads) was conducted at the Genomics Service Unit, Ludwig-Maximilian’s-Universität (LMU, München, Germany) and marker development followed established workflows [27,[34], [35], [36]]. The quality control step kept the sequences containing five repeats for penta- and tetranucleotides; eight repeats for trinucleotides; and 10 repeats for dinucleotides. Primer design (Table S2) and initial testing followed the workflow previously established [27,[34], [35], [36]]. The 36 successful primers (i.e., those that amplified a fragment of the expected size) were combined at a final concentration of 1 μM in four multiplex mixes, each with nine primer pairs (Table S2). Primers Bry1_CATA and Bry2_TATG were designed from reference library BRY16; the remaining primers were designed from reference library BRY141 (Table S2). Amplicon library preparation was conducted following Curto et al.'s [34] protocol, consisting of a first multiplex PCR followed by the pooling of all reactions per sample, PCR cleanup, and index PCR using dual barcode and custom indexing primers whose sequences are based on the TruSeq technology. Details on the workflow can be consulted in Baptista et al. [27]. Illumina MiSeq sequencing (2 x 300 bp) of indexed amplicon pools was carried out at the Genomics Service Unit at the LMU. Quality control of demultiplexed reads and merging of overlapping forward and reverse reads followed the workflow in Baptista et al. [27]. Genotype calling was achieved using the SSR_GBAS_pipeline scripts [34,35], available at https://github.com/mcurto/SSR-GBS-pipeline, generating a codominant matrix with alleles based on whole sequence information and not only amplicon length.

### 2.3 Population structure analyses

The codominant matrix resulting from the SSR-GBAS genotyping was used as input for descriptive population genetic analyses and assessment of genetic structure patterns. To avoid biases in the analyses, individuals and loci with more than 50 % missing data were excluded from the dataset. The frequency of null alleles in each marker per species was determined with FreeNA [37].

The genetic structure of the newly developed SSR markers was first assessed with a principal coordinate analysis (PCoA), colour-coded by species and geographical localities, as implemented in GenAlEx v6.51 [38,39] that allows for an evaluation of genetic structure without assumption of the Hardy-Weinberg equilibrium (HWE). Genetic structure was further investigated with STRUCTURE v2.3.4 [40,41], which assigns individuals to hypothetical populations and subpopulations by optimizing for HWE [42]. A total of 500,000 MCMC generations were performed, with an initial burn-in of 100,000 generations. Simulations considering a cluster number (*K*) between 2 and 15 were conducted, producing 10 iterations per *K*, keeping the default settings from the admixture model and allele frequencies correlated. The online pipeline CLUMPAK [43], available at http://clumpak.tau.ac.il/, was used to summarize replicates per *K*-value and infer the value for optimal detection of the suitable assumed population size for the dataset based on the delta-*K* (Δ*k*) method. Hierarchical assignment of microsatellite genotypes, based on the SSR-GBAS dataset, and further evaluation of genetic structure was achieved with the reconstruction of a network using Splitstree4 v4.18.3 [44], following the UPGMA and Consensus Network methods, based on pairwise genetic distance for codominant datasets as implemented in GenAlEx v6.51.

### 2.4 Genetic diversity indices

Genetic diversity patterns across the archipelago were evaluated using both mitochondrial and SRA-GBAS loci. The mitochondrial dataset consisted of *Reteporella* sequences of the marker cytochrome *c* oxidase subunit 1 (COI) publicly available at GenBank (www.ncbi.nlm.nih.gov/genbank) (Table S1). The COI alignment was constructed using the Clustal Omega algorithm hosted by EMBL-EBI web services [45]. For each *Reteporella* species (*sensu* [20]), genetic diversity indices in the COI dataset – number of haplotypes, haplotype and nucleotide diversity – and the SSR-GBAS dataset – number of alleles and heterozygosity levels – were estimated in DnaSP v6 [46] and GenAlEx v6.51, respectively. GenAlEx v6.51 was also used to estimate deviations from HWE in the SSR-GBAS dataset, as well as hierarchical analyses of molecular variance (AMOVA; [47]) to simultaneously assess pairwise differentiation among species and sampling localities, considering all the specimens occurring in the area regardless of their taxonomic assignment (ϕ_ST_ in the COI dataset, F_ST_ in the SSR-GBAS dataset).

## 3 Results

### 3.1 SSR-GBAS dataset

The MiSeq runs produced 18,172 and 435,151 paired reads for BRY16 (*R. atlantica*) and BRY141 (*Reteporella* sp. 6), respectively, after quality control. A total of 16,444 and 355,080 merged reads, respectively, were screened for SSR motifs. Of these, respectively, 202 and 5684 reads contained SSR motifs that complied with the criteria defined for the SSR search pipeline, resulting in 89 di-, 38 tri-, 11 tetra-, and 0 pentanucleotide repeats for BRY16; 2461 di-, 1115 tri-, 313 tetra-, and 10 pentanucleotide repeats for BRY141. A total of 42 primer pairs for *Reteporella* were designed from sequences containing penta-, tetra-, and trinucleotide repeats. Six of these failed to amplify in the single PCR test phase. Information on the remaining 36 primers, included in the four multiplex mixes is provided in Table S2.

For SSR-GBAS genotyping, a total of 16,125,228 paired reads were produced, which were reduced to 2,614,915 after quality control, merging and primer demultiplex steps. The number of reads per marker ranged from 3792 (Bry1_CATA) to 303,491 (Bry32_TTG), whereas the number of sequences generated per sample varied from 159 (BRY15 – *R. atlantica*, and DR2B2C – *Reteporella* sp. 7) to 102,359 (P8 – *R. atlantica*). The matrix was filtered for the removal of samples with more than 50 % of missing data across all markers, reducing it to 40 samples (out of 83), as follows: 20 *R. atlantica* (out of 42), three *R. tristis* (out of 14), two *R. oceanica* (out of two), seven *Reteporella* sp. 6 (out of seven) and eight *Reteporella* sp. 7 (out of 16). Although successful in the single PCR test, the markers Bry18_ATAC, Bry25_TGT, Bry27_ATA, Bry34_AAT, and Bry40_ACT yielded more than 50 % missing data and were excluded from the SSR-GBAS dataset. A total of 31 markers in the SSR-GBAS dataset were suitable for population genetic analyses, with the number of alleles ranging from 15 (Bry26_TATG, Bry26_ATA) to 66 (Bry8_TGTC) (Table S2). Groups with less than five individuals were not considered for population-based analyses, such as genetic diversity and differentiation indices, ensuring the reliability of the analyses.

**Fig. 3:**
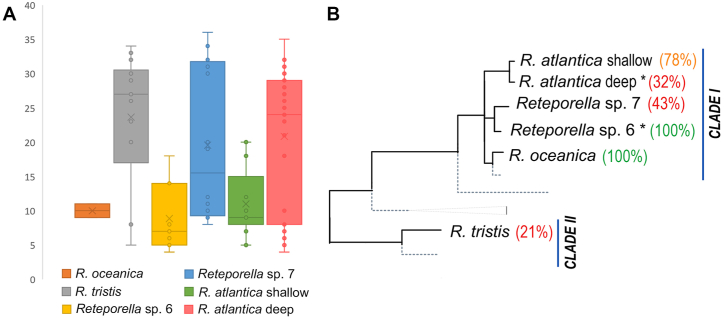
Efficiency of the SSR-GBAS markers designed for Azorean *Reteporella* species. **A)** Missing data, given in absolute numbers of primer pairs that failed amplification. Internal points represent missing data per individual sample; *Reteporella* species are colour-coded. **B)** Proportion of samples of each *Reteporella* species amplified with less than 50 % missing data and selected for further analyses: red (<50 %), orange (50–85 %), green (>85 %). Asterisk (∗) indicates the species used in primer design (*R. atlantica* – two primer pairs, *Reteporella* sp. 6–29 primer pairs). Grey dotted branches represent *Reteporella* species not included in the SSR-GBAS analysis. Phylogeny adapted from Baptista et al. [20]. (For interpretation of the references to colour in this figure legend, the reader is referred to the Web version of this article.)

Marker efficiency varied across the five *Reteporella* species studied (Fig. 3). Missing data per sample, considering the unfiltered dataset, was widely variable in *Reteporella* sp. 7 and *R. atlantica;* high levels of missing data were inferred for *R. tristis*; marker failure was low in *Reteporella* sp. 6, *R. oceanica,* and shallow-water *R. atlantica* (Fig. 3A). The SSR-GBAS primer set performed better in species assigned to Clade I and worse in species with a higher incidence of missing data. This is suggested by the proportion of samples for each *Reteporella* species amplified with less than 50 % missing data and selected for downstream analyses (Fig. 3B): all samples of *Reteporella* sp. 6 and *R. oceanica*, 43 % of *Reteporella* sp. 7 samples. Only 21 % of *R. tristis* (Clade II) specimens were successfully amplified. A discrepancy in the amplification success of *R. atlantica* from shallow- and deep-waters was observed, with higher success in the former (78 %).

### 3.2 Population structure of *Reteporella* species across the Azores archipelago

**Fig. 4:**
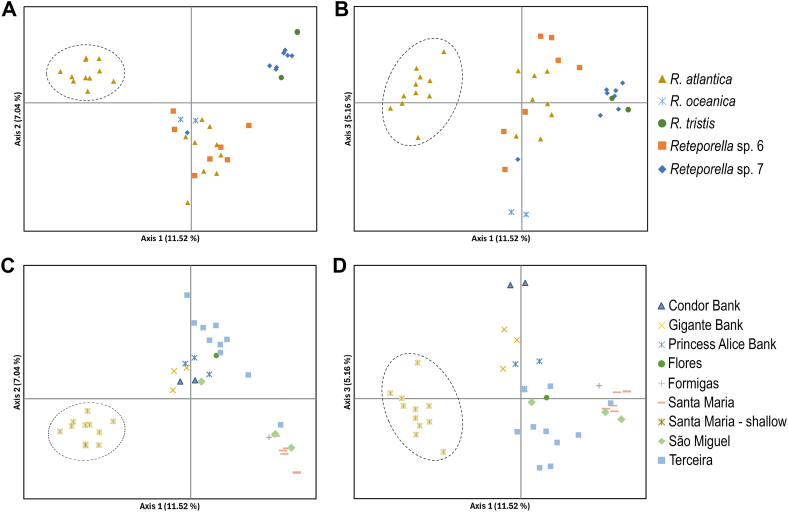
Principal coordinates analyses based on the SSR-GBAS dataset of *Reteporella* from the Azores. Analyses partitioned by species (**A-B**) and geographical localities (**C-D**). **A)** Variance explained by axis 1 (11.52 %) and axis 2 (7.04 %) in the analysis by species; **B)** Variance explained by axis 1 (11.52 %) and axis 3 (5.16 %) in the analysis by species; **C)** Variance explained by axis 1 (11.52 %) and axis 2 (7.04 %) in the analysis by locality; **D)** Variance explained by axis 1 (11.52 %) and axis 3 (5.16 %) in the analysis by locality. Cluster of *R. atlantica* from shallow waters of Santa Maria Island (<25 m) delimited with dash-lines. PCoAs conducted in GenAlEx v6.5 [38,39]; groups in study are coded by colour and icon shape. (For interpretation of the references to colour in this figure legend, the reader is referred to the Web version of this article.)

PCoA analysis of the complete SSR-GBAS dataset with samples colour-coded by species (Fig. 4A and B) showed a clear differentiation of a *R. atlantica* group composed exclusively of shallow-water specimens (<25 m) from around Santa Maria. Most of the *Reteporella* sp. 7 individuals form a genetically distinct cluster with *R. tristis*. Although positioned closely with *R. tristis,* this does not indicate phylogenetic resemblance between the taxa [20]. The remaining species/populations did not form identifiable clusters in any of the three dimensions. The PCoA analysis was repeated after removal of the shallow-water *R. atlantica* specimens, aiming to clarify the relationships among the remaining taxa, but no further clustering was obtained (Fig. S1). The PCoA, plotted by geographical locations (Fig. 4C and D), also supports a clear distinction of samples from shallowwaters (<25 m) around Santa Maria in every dimension. Overall, samples of the same locality clustered closely. When comparing axes 1 and 2, some geographical clustering can be distinguished: mostly eastern (Santa Maria and São Miguel) localities in the bottom right corner, Terceira samples were distributed in a linear pattern in the top right quadrant of the plot, and the seamounts tended to cluster in the centre of the plot.

**Fig. 5:**
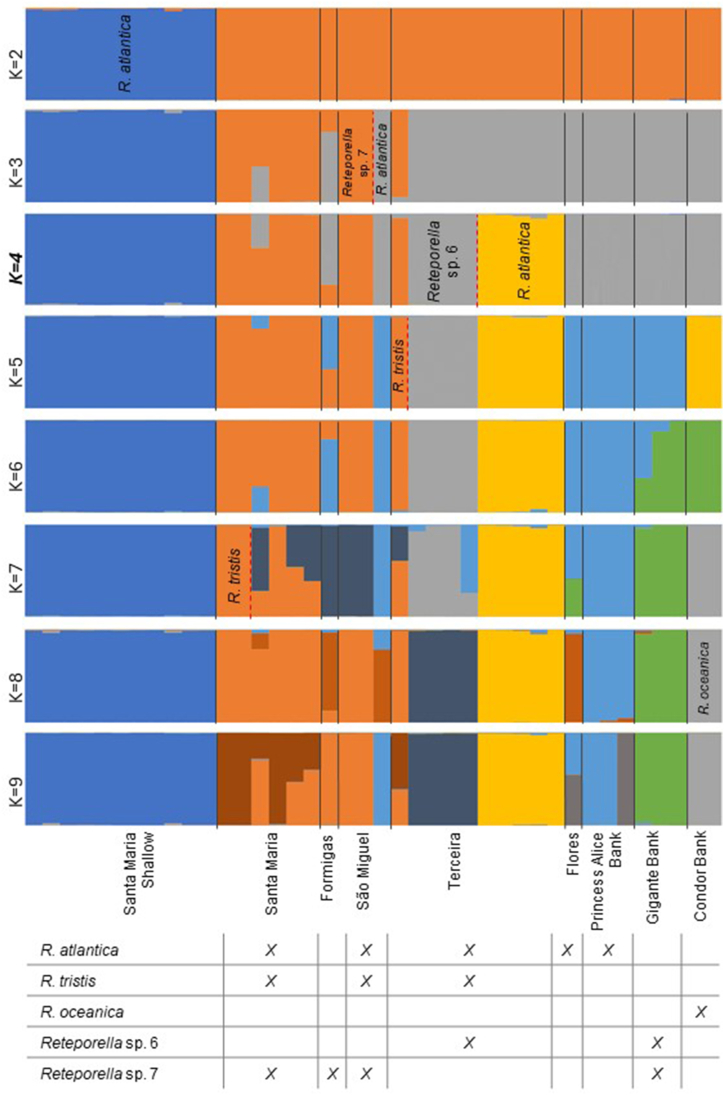
Genetic structure analysis of the complete SSR-GBAS dataset of *Reteporella* and species present at each geographical locality. Inferred with STRUCTURE v2.3.4 [40,41] and reporting the results of *K*-values from 2 to 9, with an optimal *K* = 4. As K increases, the software progressively assigns individuals of the same species to a single cluster (within each locality). Species names are shown in the plot whenever they are distinguished as single clusters within a locality for the first time. Black lines divide the localities, whereas the dashed red lines indicate the subdivision into taxon-specific clusters, within each locality, as *K* increases. The table reports the species present at each locality and successfully included in the SSR-GBAS dataset. Please note that the geographical distribution of these species is broader than reported in the table and can be consulted elsewhere [20]. (For interpretation of the references to colour in this figure legend, the reader is referred to the Web version of this article.)

**Fig. 6:**
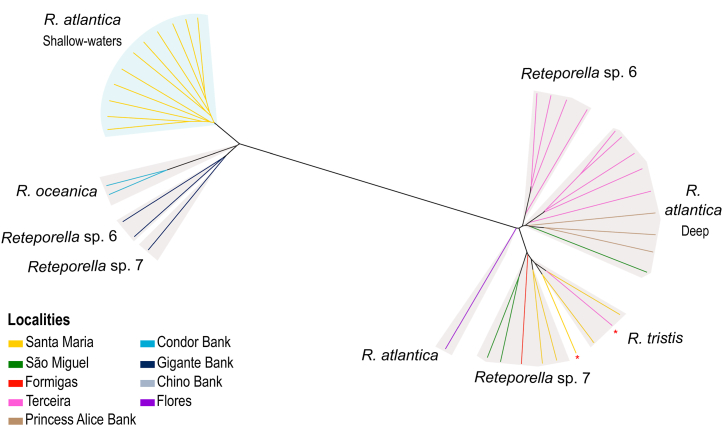
Genetic distance network of Azorean *Reteporella* species, based on the SSR-GBAS dataset, representing the hierarchical assignment by geographical locality and further subdivision by species. Reconstructed with Splitstree4 v4.18.3 [44] under the UPGMA method. Branches color-coded by geographical locality as in the bottom left corner. Clusters of the same species are identified and delimited in light brown. Red asterisks (∗) indicate outlier specimens in species or locality assignment. (For interpretation of the references to colour in this figure legend, the reader is referred to the Web version of this article.)

In the STRUCTURE reconstruction (Fig. 5), four genetic clusters were defined as optimal and shared by different *Reteporella* species that co-exist in the same deep-water localities, as all specimens were collected from depths over 150 m (see Table S1), except for the shallow-water samples from Santa Maria. Within *R. atlantica,* a genetic group comprising only the shallow-water specimens of Santa Maria emerged at *K* = 2 and was consistent throughout the *K*-values tested. In general, the major clusters matched the geographical distribution of the samples: shallow-water *R. atlantica* from Santa Maria, *R. atlantica* from Terceira, one predominant in deep waters of Santa Maria and São Miguel (eastern group), and another from the seamounts and Terceira (central group). For *K* = 4, two clusters were inferred in São Miguel (*Reteporella* sp. 7 and *R. atlantica* – deep), whilst in Terceira we can distinguish three genetic groups (*Reteporella* sp. 6, *R. tristis*, and *R. atlantica* – deep). Population structure became more complex with higher *K*-values, as each locality was subdivided into clusters mainly corresponding to the species occurring there, however with limited admixture with conspecifics in other localities. At *K* = 7, a cluster of *R. atlantica* was shared among São Miguel and Princess Alice Bank. *Reteporella oceanica* from Condor Bank, formed an independent cluster at *K* = 8. The clustering analyses were not biologically informative for *K*-values over 9 (data not shown). The reconstruction of a genetic distance network (Fig. 6) supported the hierarchical assignment of microsatellite clusters by showing deeper splits based on geographical localities and shallower by species present in the area.

**Fig. 7:**
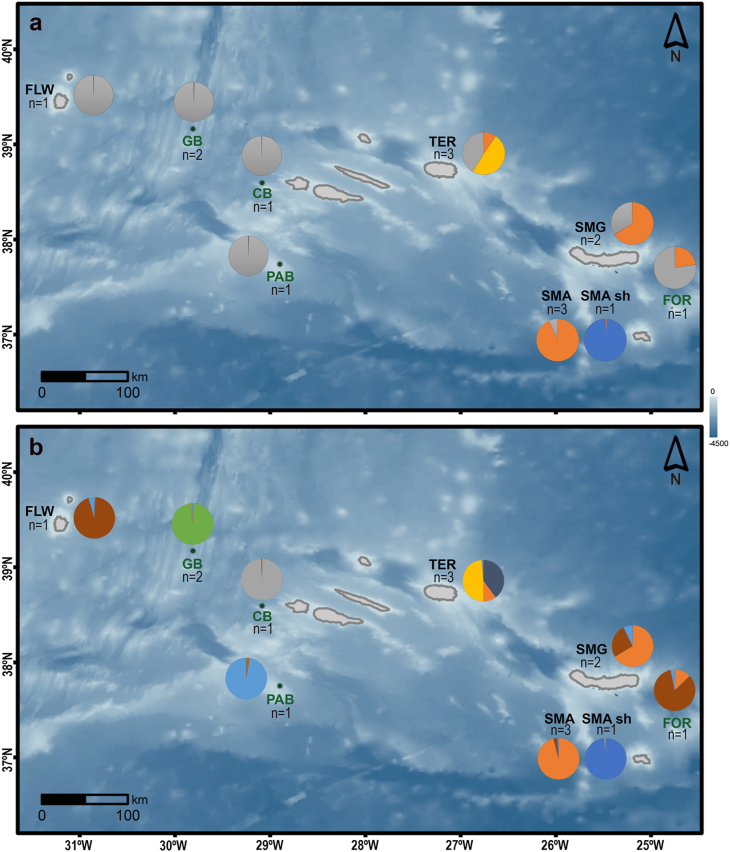
Directional gradient of the frequency of each genetic cluster per geographical locality, estimated in the STRUCTURE analysis. A) For optimal *K* = 4; **B)** For the maximum informative *K* = 8. Number of species (n) known to occur at each locality is indicated under the locality designation. Pie chart colours correspond to the clusters at *K* = 4 and *K* = 8 (Fig. 5). Abbreviations are depicted in black for islands (SMA – Santa Maria, SMG – São Miguel, TER – Terceira, FLW – Flores) and green for seamounts (FOR – Formigas, PAB – Princess Alice Bank, CB – Condor Bank, GB – Gigante Bank). Coastline shape retrieved from the Portuguese Hydrographic Institute (https://www.hidrografico.pt/op/33) and bathymetry, indicated at the right margin, derived from GEBCO 2020 (https://www.gebco.net/data_and_products/gridded_bathymetry_data/). (For interpretation of the references to colour in this figure legend, the reader is referred to the Web version of this article.)

By plotting STRUCTURE cluster assignment onto a map, a longitudinal gradient of shared clusters in the Azores was observed (Fig. 7). For example, for *K* = 4, a cluster (grey), despite being found all over the archipelago, dominates in the west being shared by all individuals of Flores and the central seamounts; a cluster (orange) is only found in Santa Maria, São Miguel, Formigas, and Terceira being more prevalent in the east; the remaining clusters are island-specific, comprising the shallow waters of Santa Maria island (blue) and some individuals of Terceira island (yellow). For *K* = 8, the clusters are geographically more restricted and often a single haplotype dominates (e.g., almost-exclusive genotypes in the westernmost localities, two haplotypes typical of Terceira, and shared diversity in the easternmost localities).

### 3.3 Genetic diversity indices

Genetic diversity indices, summarized in Table S3, were estimated for species using both the COI and SSR-GBAS datasets. Overall, haplotype and nucleotide diversity were high (≥0.700 and ≥0.00848, respectively) across the four species analysed with the COI marker. *Reteporella atlantica* was represented by the largest number of individuals (53) and yielded the highest haplotype diversity (0.858). Similar levels of haplotype diversity were obtained for *Reteporella* spp. 6 and 7, which were represented by fewer individuals, i.e., 7 and 18, respectively. *Reteporella tristis* had the lowest haplotype diversity (0.7), with only four haplotypes occurring across 16 individuals. Overall, haplotype diversity was high across taxa, except in *R. atlantica* of shallow waters (0.00058). Heterozygosity levels in the SSR-GBAS dataset were estimated without accounting for monomorphic markers, revealing lower observed heterozygosity than expected in all the species analysed (≥5 individuals).

Deviations from HWE per microsatellite locus were estimated for each species. The cause of the deviations was assessed by estimating the frequency of null alleles and checking the original matrix for homozygote excess. HWE deviation results are summarized alongside the frequency of null alleles in Table S4. A high incidence of null alleles was detected at several loci, although never consistently across all taxa. Significant deviation from HWE was shown at seven loci in *Reteporella* sp. 6 and eight loci of *Reteporella* sp. 7. However, the highest level of deviation from HWE was observed in *R. atlantica* (26 out of 31) with a high frequency of null alleles associated with most of them. The number of loci deviating from HWE decreased when separating deep- and shallow-water *R. atlantica* samples, but the shallow-water cluster still revealed a large number of deviations (13).

### 3.4 Differentiation amongst *Reteporella* species in the Azores

The AMOVA by species, based on the SSR-GBAS dataset, revealed that only 11 % of the genetic variation can be explained by the current classification scheme (Table S5a). The AMOVA analysis by geographical localities (Table S5b), regardless of species assignment, attributed only 17 % of the genetic variation among localities, a value that was reduced to 14 % after removing the shallow-water samples from Santa Maria (Table S5c). In all cases, over 38 % of the variation in the SSR-GBAS dataset occurred among individuals. The AMOVA analyses were repeated by each locus per species and localities to assess whether the structure pattern retrieved by the SSR-GBAS dataset was an artefact of a single or few loci (Fig. S2). For most markers (>18), up to 25 % of the genetic variation was consistently explained by partition in species or localities; outliers were not detected. The matrices of markers with over 25 % of genetic variation in the dataset were checked and it was verified that the levels of variation are likely related to the marker genotyping success, especially in shallow-water *R. atlantica* (data not shown). Regarding F-statistics, significant (*p* < 0.05) F_ST_ values of 0.172 and 0.136 were estimated for the overall SSR-GBAS dataset by locality with and without shallow-water samples, respectively. In the COI dataset, the variation among species accounts for 83 %, whilst among geographical localities this value decreases to 43 % (Table S6). Regarding ϕ-statistics, significant (*p* < 0.05) ϕ_PT_ values of 0.825 and 0.433 were obtained per species and geographical localities, respectively.

## 4 Discussion

In this work, we aimed at understanding the dynamics shaping the evolution of bryozoans in the remote Azores Archipelago and shedding some light on evolutionary processes and patterns that are probably more widespread in the marine realm than previously thought. Phylogenetic reconstructions [20] support the occurrence of at least eight *Reteporella* species in the Azores. Microsatellite data of five *Reteporella* species, based on the first application of the SSR-GBAS approach on bryozoans, suggests the occurrence of interspecific genetic exchanges in regions of sympatry. Nevertheless, we cannot entirely exclude the possibility that this signal is an artefact of the methodology used, based on the amplification of multiple loci (see section 4.2). A potentially cryptic lineage of *R. atlantica* seems to occur only in shallow waters of Santa Maria Island, presumably as a consequence of ecologically-driven differentiation across its bathymetrical range.

Unexpected processes and patterns of diversification are uncovered as population genetic studies are progressively being applied to address the diversity and population dynamics of lesser-known marine invertebrates [48,49]. This study builds on the current knowledge of the few population genetic studies on bryozoans, which are restricted to predominantly invasive taxa [5,6].

### 4.1 Performance of SSR-GBAS markers

Disparity in the performance and success of the SSR-GBAS primers is expected in a situation of cross-species amplification, due to allele dropout comparable to other amplification based methods [34,50]. It is related to the phylogenetic relationships of the studied taxa due to the so-called “ascertainment bias” [51], as better rates of success were obtained for taxa that are closely related to those used to design the primers from (*R. atlantica* and *Reteporella* sp. 6). As only two primers were designed from *R. atlantica*, the success rates in this species are lower than those estimated for *Reteporella* sp. 6. Cross-species amplification can also be locus-dependant and affected by admixture [35], as detected in this study. Previous tests of cross-species amplification using SSR-GBAS primer sets showed that the development of markers based on several species minimises the ascertainment bias within each of the study species, whilst using only one marker set from one species would contribute to biases in estimated variability values [34]. Designing primers for each species was, however, not possible within the framework of this study.

### 4.2 Discordance between marker classes

The adjoined use of COI and SSR-GBAS datasets is a powerful tool to evaluate different inheritance dynamics and processes that shape phylogeographic and demographic patterns [52]. Mitochondrial markers provide insights into the initial colonization and phylogenetic evolution, whereas microsatellite markers allow the assessment of fine-scale genetic structure and population fragmentation, as well as the detection of signatures of reticulate events such as hybridization in recent times [[52], [53], [54]]. All species studied (*R. atlantica*, *R. oceanica, R. tristis*, and *Reteporella* spp. 6 and 7) are morphologically distinguishable, with COI divergence levels over 6 % [20] and high genetic diversity levels as inferred in this study suggesting clear interspecific divergence. However, discordant cytonuclear patterns were inferred in these species. Baptista et al. [20] reconstructed haplotypic networks based on COI, retrieving a haplotype structure congruent with geographical distribution for *R.* atlantica, *R. tristis* and *Reteporella* spp. 6 and 7; each species formed a distinct network cluster, supporting species delimitation. In contrast, the genetic structure inferred with the SSR-GBAS dataset was less pronounced and some COI haplotypes are shared by different genotype clusters. Such discordance was unexpected, as the faster rate of evolution of microsatellite markers [53] should result in a strong population structure in *Reteporella* species, as predicted under the hypothesis being tested.

As mentioned before, primer pairs designed from *R. atlantica* and *Reteporella* sp. 6 were applied to a dataset comprising five *Reteporella* species, in a typical case of cross-species amplification. Mutations in the primer-binding region are expected across different taxa, with the potential to cause allelic dropout and to prevent the amplification of more divergent alleles [34,50,55]. For different species, the same loci will be amplified with different rates of efficiency, causing disparity in the performance of the primer battery: null alleles will arise differently across different species, while the amplification of alleles shared across species should not be affected [34,50,55]. This might result in an underestimation of genetic differentiation and structure patterns that resemble null allele distribution rather than genetic similarity in the microsatellite evolution. The weak genetic structure inferred for the genus *Reteporella* (except for shallow-water *R. atlantica*; Fig. 4, Fig. 5, Fig. 6, Fig. 7) with the SSR-GBAS is proposed to be due to methodological constraints inherent to the characteristics of multilocus approaches based on amplification, as a consequence of null allele distribution and disparity in the marker performance in the five species studied. Because the amount of allele dropout causing null alleles in the analysis can be assumed to be higher for more variable loci, the genetic distance between the genotype clusters, as indicated in the COI network analysis [20], can be underestimated. In this case, variable loci will rather provide information for within-species variation and not for interspecific differentiation. The distances between the multilocus and COI haplotype datasets are thus not directly comparable. The overall structure between the species inferred by the SSR-GBAS dataset generally supports the species concept inferred by COI and morphology [20].

### 4.3 Population structure in the Azores Archipelago

Regardless of the constraints of multilocus methods for cross-species investigations, the congruence detected between nuclear patterns and the geographical distribution of specimens was striking. The SSR-GBAS dataset suggests a first subdivision by geographical locality and then by species assignment within each locality (cf. *K* > 4 in Fig. 5, Fig. 6, Fig. 7). As an alternative hypothesis to null alleles, other dynamic processes, such as interspecific introgression in areas of sympatry, might be driving the evolution of Azorean *Reteporella*. Incongruence between mitochondrial and microsatellite/nuclear markers has been reported in other marine sessile taxa, and is suggested to be a consequence of semipermeable species boundaries or incomplete sorting of ancestral polymorphisms [[56], [57], [58], [59]]. Occasional gene flow, involving mostly neutral nuclear regions, favours locally dependent exchanges between closely related species co-occurring in one locality, rather than an exchange between conspecific colonies located far apart [56,60]. Such exchanges might be kept in an interplay with the selective forces acting upon the mitochondrial DNA, maintaining the morphological integrity and phylogenetic identity of each species [53,56,[61], [62], [63]]. Testing this hypothesis is, unfortunately, not possible with the available dataset and because of a low sample size for each species and locality (see section 4.6).

### 4.4 Genetic diversity in *Reteporella* species

Both primer pairs designed from a *R. atlantica* library (BRY16) showed significant deviation from HWE only in the target species. Mitochondrial and morphological data (not shown) suggest that this specimen might constitute a potentially divergent lineage of deep-water *R. atlantica* [20]. If this is confirmed in future integrative analyses, lower specificity of primers even for closely related *R. atlantica* populations can explain the deviations observed [50,55].

Observed heterozygosity was lower than the expected heterozygosity in all *Reteporella* species studied. Life traits may have contributed to the deviation from HWE and confounded the population genetic signals. In *Reteporella* species and other cheilostomes, dispersal occurs either via short-lived, non-planktotrophic larvae or by rafting of adult, bisexual colonies. The limited dispersal capacity of the larvae, together with the hydrographic conditions in the archipelago, likely contribute to the increasing isolation and divergence of distant populations [27,32,64,65]. Subpopulation structure contributes to an expected deficit of heterozygotes and an apparent excess of homozygotes, as inferred for all *Reteporella* species which can be attributed to the Wahlund effect [66,67]. Sex biases can also contribute to deviations from HWE. Bryozoan colonies are not distinguished by sex, producing both male and female gametes, thus sex-biased dispersal [52] is unlikely to affect HWE in the genus *Reteporella*.

High effective population sizes have also been shown to cause deviations from the HWE [52]. Due to the high amount of missing data and evidence of null alleles, estimates of effective population sizes based on our dataset would be biased, such that its effect on population structure cannot be accounted for. Most loci amplified of *R. atlantica* deviate from HWE due to homozygote excess or, particularly, a higher incidence of null alleles in the shallow waters of Santa Maria (eastern group of islands). The lower efficiency of the SSR-GBAS primers in the shallow-water cluster was expected as these populations appear to constitute a genetically distinct lineage of *R. atlantica* [20]. Most *R. atlantica* specimens from Flores (western group of islands) failed to amplify with the SSR-GBAS primer battery due to poor quality of the DNA or high sequence divergence in the primer annealing positions [34,50] if these constitute a divergent deep-water lineage. Heterozygote genotypes erroneously genotyped as homozygotes can explain the deficit of heterozygotes [68] inferred for almost all *Reteporella* species, although it is unlikely considering the workflow and respective checkpoints.

### 4.5 The cryptic *Reteporella atlantica* lineage in shallow waters

Shallow-water populations of *R. atlantica* from the easternmost island of Santa Maria stand out in all the analyses as different from conspecifics, especially in the SSR-GBAS dataset, as a shallow-water cluster is constantly retrieved across the inferences.

*Reteporella atlantica*, which was originally described from the Faial-Pico Channel, is found around Santa Maria at diving depths (<25 m), mostly in low energy conditions on the ceilings and upper sidewalls of submerged caves or tunnels, protected from wave action and with low luminosity (LB and BB, pers. obs., 2019–2023). Sampling was conducted in July 2019 (Summer) at depths of 11–28 m and seawater temperatures of 18–19 °C. Although similar environments occur in other Azorean islands, *R. atlantica* colonies were not observed in shallow waters elsewhere, according to reports of experienced and knowledgeable divers, as well as our field observations (LB, pers. obs., July and August 2019–2023). The next shallowest record is from a settlement panel deployed at 60 m depth off Faial [69], which is above the storm wave base at around 100 m in the archipelago. In the settlement panel experiment [69], *Reteporella* colonies grew to a height and width of *c.* 2 cm within a year of submersion at 60 m depth and to about 1 cm at 150 m [70]. Colonies may exceed a size of 10 cm, being prone to abrasion in high-energy environments during their >10 years’ lifetime, so it is expected that larvae preferentially settle in calm conditions to reduce the exposure to unfavourable environments. Thus, the greatest diversity of *Reteporella* species in the Azores and elsewhere is found below the storm wave base. None of the other Azorean species occur in depths shallower than 150 m [20]. In contrast, numerous *Reteporella* species occur in various shallow-water environments in the Mediterranean Sea, in which the fair-weather and storm wave bases are considerably shallower [71].

Nevertheless, the absence of *R. atlantica* from sheltered, shallow-water environments in Azorean islands other than Santa Maria is striking. Santa Maria is the easternmost island of the archipelago where sea-surface currents flow in a prevailing eastward direction [21]. Therefore, larvae of the shallow-water *R. atlantica* are likely lost to the open ocean, hampering the colonization of the more westerly positioned islands. Moreover, ocean depth has been shown to limit dispersal in shallow water organisms with short-lived or no juvenile larvae, acting as a barrier even at short geographic distances within oceanic volcanic archipelagos [72]. Thus, if the group remains reproductively isolated, the Santa Maria's population of *R. atlantica* may eventually evolve into a single-island marine endemic species, which are rarely recorded in the marine realm [[73], [74]].

Morphologically, specimens from shallow waters of Santa Maria match the taxonomical concept of *R. atlantica* [18], but the COI and SSR-GBAS datasets showed a clear distinction of the group with no evidence of introgression with other *Reteporella* taxa from deeper waters. Unfortunately, *R. atlantica* is not represented among the 17 deep-water specimens collected off Santa Maria (see Table S1), thus it was neither possible to ascertain the levels of divergence between shallow- and deep-water lineages in this area, nor to determine the occurrence of gene flow with other localities at the same depth. The shallow-water *R. atlantica* populations might constitute an example of potentially cryptic diversity among Azorean bryozoans, with ecological differentiation preceding a phylogenetic split [75,76]. In this case, the two lineages have recently adapted to different environmental conditions (shallow vs. deep waters) but reproductive isolation might not yet be complete. With the current dataset, we cannot determine the existence of reproductive barriers between the lineages.

Bathymetrical zonation might play a role in promoting the differentiation of the shallow-water lineage of *R. atlantica* [60,77], as already reported in several other marine sessile organisms (corals in Refs. [56,76,78,79]; review by Knowlton [80]). The low tendency for vertical migration of larvae and/or adults contributes to the divergence of lineages to prevail over the vertical rather than horizontal axis in other marine sessile invertebrates [49,56,81]. Regional and local hydrographic patterns are also known to affect gene flow and species distribution in these [64,75,82]. Thermocline position, estimated between 40 and 100 m in the Azores region [22], often acts as a barrier to populations at different depths due to a light, nutrient, and temperature gradient [83], with changes in predation pressure, competition for space, as well as currents as depth increases [56,78]. Reproductive isolation between deep- and shallow-water colonies can be maintained by pleiotropic interactions, such as mismatching reproductive timings as reported for *Eunicea flexuosa* [76,78,84].

### 4.6 Future research and recommendations

Despite the differences in resolution on the species level between the SSR-GBAS and COI datasets, the analyses support the five *Reteporella* taxa distinguished by morphological characters and mitochondrial differentiation. Future work will investigate to what extent constraints in cross-amplification of the markers or interspecific introgression in areas of sympatry explain the discordance between the datasets. Phylogenomic approaches with a comprehensive *Reteporella* dataset might be useful for testing the hybridization hypothesis as a non-neutral process and identifying putative genomic regions of congruence among species. These approaches could also be used to evaluate possible loci duplications or unreported polyploidy with the potential to mask phylogeographic and population genetic patterns. The genetic architecture and mechanisms acting on bryozoan genomes are far from fully comprehended. Recently, the largest number of mitochondrial introns ever recorded from bilaterian animals were described in four bryozoan species [85], and substantial chromosome rearrangements seem to have shaped the evolution of bryozoan genomes [86]. Furthermore, large-scale bryozoan phylogenies at higher systematic levels are only now becoming available from next-generation sequencing techniques [1,2]. Future phylogenomic approaches can assess to what extent the evolution of *Reteporella* and other cheilostomes is shaped by dynamic genomic processes.

Two alternative scenarios, which await testing in the future, are proposed to explain the finding of a potentially cryptic ecotype of *R. atlantica* restricted to shallow waters around Santa Maria. For the “bottom-up” scenario we suggest that the archipelago was colonised from other nearby deep-sea source regions such as the Mid-Atlantic Ridge [87] or the cluster of seamounts around the Great Meteor Bank south of the Azores where the genus is present (BB, pers. obs.), and that, at some time, an opportunity arose for *R. atlantica* to invade shallower niches. A recent divergence and reproductive isolation of this group could explain its low nucleotide and haplotype diversities (Table S3). As Santa Maria is the oldest island in the archipelago, ample time was potentially available for the island’s population to adapt to initially adverse shallow-water conditions (e.g., higher current energy and abrasion rate, competition with faster-growing photoautotrophs, among others). This scenario, however, would have likely taken place also in other Azorean islands given their geological age and because *Reteporella* species seem to be successful across wide bathymetrical and environmental ranges, with different species occurring in sympatry [20].

Alternatively, in the “top-down” scenario, the shallow-water *R. atlantica* would represent the ancestral lineage (at least of Clade I) that first colonized the Azores. The most plausible mechanism of transport of brooding bryozoans, such as *Reteporella*, to the remote Azores Archipelago is by rafting of adult colonies from shallow water source populations on floating substrata [31,32,88,89]. Following the subsequent origin of the other islands, the deeper population of *R. atlantica* may have established itself throughout the remaining archipelago.

Whether the shallow-water lineage constitutes a recent divergence restricted to Santa Maria or is a relic of an ancestral shallow-water source population remains to be tested. A better understanding of the colonization history of the Azores would greatly benefit from a phylogenetic reconstruction of the family Phidoloporidae in the North Atlantic.

## 5 Conclusions

This study constitutes the first application of a genotyping approach like SSR-GBAS on bryozoans, revealing potential signatures of events shaping the evolution of the genus *Reteporella* in the remote Azores Archipelago. The biogeographical analyses derived from mitochondrial and microsatellite markers retrieved incongruent patterns. The biological meaning of these genetic patterns, reflecting geographical distance and species boundaries, might indicate the occurrence of interspecific hybridization among *Reteporella* species in deep waters. Moreover, we detected the existence of a shallow-water lineage of *R. atlantica*, likely undergoing ecologically-driven incipient speciation, which raises new questions regarding the biogeography of sessile marine organisms across their bathymetrical range. Marine invertebrates are often overlooked during the implementation of conservation and management strategies. The finding of cryptic lineages in shallow waters and possible hybridization in deep habitats suggest that these areas might be crucial for the dynamics and resilience of unique oceanic insular ecosystems. The patterns herein reported might be more widespread than previously thought across the phylum Bryozoa, and the extent of ecological differentiation and hybridization in marine animal species is now starting to be unveiled, resulting in important evolutionary insights. A better understanding of how genomic dynamics affects the evolution of *Reteporella* and other cheilostomes depends upon advances in basic molecular knowledge of bryozoans and the future application of phylogenomic approaches.

## Data accessibility statement

Raw reads of the two shot-gun sequencing runs obtained for *Reteporella* for marker discovery*,* as well as the raw reads for the SSR-GBAS runs can be accessed at the GenBank short read archive, BioProject number PRJNA1130121. The mitochondrial COI data analysed in this article were retrieved from GenBank (accession numbers OP086099-OP086147; OP086149-OP086152; OP086154-OP086181; OP086183-OP086198).

## Ethics statement

This work does not involve animal experiments or human studies. Fresh specimens were collected during the scientific cruise M150 “Controls in benthic and pelagic BIODIversity of the AZores BIODIAZ” with the German RV *Meteor* in 2018 (George et al., 2018), with respective permits. Prospection and sampling by SCUBA diving around Santa Maria Island were authorized by Direção Reginal da Ciência e Tecnologia, Governo Regional dos Açores under the permits CCIP 24/2019/DRCT and 29/2019/DRCT. Material deposited at biological collections (DBUA – Marine Molluscs Collection of the Department of Biology of the University of the Azores and DOP – Department of Oceanography and Fisheries of the University of the Azores) were loaned with the permission of the curators.

## CRediT authorship contribution statement

**Lara Baptista:** Writing – original draft, Visualization, Methodology, Investigation, Formal analysis, Data curation, Conceptualization. **Manuel Curto:** Writing – original draft, Visualization, Validation, Methodology, Conceptualization. **Andrea Waeschenbach:** Writing – review & editing, Visualization, Validation. **Björn Berning:** Writing – review & editing, Writing – original draft, Visualization, Validation. **António M. Santos:** Writing – review & editing, Validation, Resources, Funding acquisition, Conceptualization. **Sérgio P. Ávila:** Writing – review & editing, Resources, Funding acquisition. **Harald Meimberg:** Writing – review & editing, Validation, Resources, Funding acquisition, Conceptualization.

## Declaration of competing interest

The authors declare the following financial interests/personal relationships which may be considered as potential competing interests: Lara Baptista reports financial support was provided by 10.13039/501100019370Foundation for Science and Technology. Sérgio P. Ávila reports financial support was provided by 10.13039/501100019370Foundation for Science and Technology. Lara Baptista reports financial support, administrative support, and travel were provided by 10.13039/100018693Horizon Europe. Bjorn Berning reports financial support, administrative support, and travel were provided by 10.13039/100018693Horizon Europe. If there are other authors, they declare that they have no known competing financial interests or personal relationships that could have appeared to influence the work reported in this paper.
