# Supplementary material for: Population genetic structure and ecological differentiation in the bryozoan genus Reteporella across the Azores Archipelago (central North Atlantic)
Source: Heliyon. 2024 Oct 1;10(19):e38765. doi: 10.1016/j.heliyon.2024.e38765 (PMC11489315; doi:10.1016/j.heliyon.2024.e38765)
Supplement: Multimedia component 1 [file mmc1.docx]

**Supplementary Material**

**Population genetic structure and ecological differentiation in the bryozoan genus *Reteporella* across the Azores Archipelago (central North Atlantic)**

**Lara Baptista**^a,b,c,d,1 *^, Manuel Curto^b,d^, Andrea Waeschenbach^e^, Björn Berning^a^, António M. Santos^b,d,f^, Sérgio P. Ávila^a,b,f,g,h ¥^, Harald Meimberg^c ¥^

^a^ CIBIO-InBIO, Universidade dos Açores, Departamento de Biologia, Rua Mãe de Deus 13A, 9501-801 Ponta Delgada, São Miguel, Portugal

^b^ BIOPOLIS Program in Genomics, Biodiversity and Land Planning, CIBIO, Campus de Vairão, Vairão, 4485-661 Portugal

^c^ Institute for Integrative Nature Conservation Research , Department of Integrative Biology and Biodiversity Research, BOKU University, Gregor-Mendel-Straße 33, 1180 Wien, Austria

^d^ CIBIO, Centro de Investigação em Biodiversidade e Recursos Genéticos, InBIO Laboratório Associado, Campus de Vairão, Universidade do Porto, Rua Padre Armando Quintas, no. 7, 4485-661 Vairão, Portugal

^e^ Science, Natural History Museum, Cromwell Road, London, SW7 5BD, United Kingdom

^f^ Faculdade de Ciências da Universidade do Porto, Rua do Campo Alegre 1021/1055, 4169-007 Porto, Portugal

^g^ UNESCO Chair – Land Within Sea: Biodiversity & Sustainability in Atlantic Islands, Universidade dos Açores, 9501-801 Ponta Delgada, Portugal

^h^ Departamento de Biologia, Faculdade de Ciências e Tecnologia, Universidade dos Açores, 9501-801 Ponta Delgada, Açores, Portugal

*Corresponding author: Lara Baptista (laracbaptista@hotmail.com); ORCID: 0000-0002-4429-9855

^¥^ Sérgio P. Ávila and Harald Meimberg should be considered joint senior author.

**Table S1.** Detailed information of Reteporella samples used in this work. Localities with coordinates in decimal degrees (when available), depth (in meters), collection voucher (if applicable), and COI GenBank accession numbers are provided. Column “SSR data” indicates samples for which short-sequence repeats were successfully amplified and analysed (**x**) or that failed amplification (f**;** less than 50 % loci successfully genotyped); n.a. indicates samples not included in the Illumina MiSeq run. BBerning-SP = personal collection of Björn Berning; DBUA = Department of Biology of the University of the Azores; DOP = Department of Oceanography and Fisheries of the University of the Azores; M150-BIOD = Controls in benthic and pelagic BIODIversity of the AZores BIODIAZ” with the German RV Meteor in 2018; SaM-ID = Senckenberg Natural History Museum

| **Species** | **Sample ID** | **Locality** | **Depth (m)** | **Coordinates DD** | **Collection voucher** | **COI Acc.** | **SSR data** |  |
| --- | --- | --- | --- | --- | --- | --- | --- | --- |
|  |  |  |  |  |  |  |  |  |
| ***R. atlantica*** | P8 | Santa Maria, Azores | 24 | 36.935, -25.092 | DBUA:BRY 009 | OP086115 | **x** |  |
|  | P10 | Santa Maria, Azores | 24 | 36.935, -25.092 | DBUA:BRY 011 | OP086116 | *f* |  |
|  | P15 | Santa Maria, Azores | 24 | 36.935, -25.092 | DBUA:BRY 016 | OP086117 | **x** |  |
|  | P16 | Santa Maria, Azores | 28 | 36.935, -25.092 | DBUA:BRY 017 | OP086106 | *f* |  |
|  | P17 | Santa Maria, Azores | 28 | 36.935, -25.092 | DBUA:BRY 018 | OP086118 | **x** |  |
|  | MB4 | Santa Maria, Azores | 27 | 36.995, -25.187 | DBUA:BRY 023 | OP086107 | **x** |  |
|  | MB5 | Santa Maria, Azores | 27 | 36.995, -25.187 | DBUA:BRY 024 | OP086104 | **x** |  |
|  | MB6 | Santa Maria, Azores | 27 | 36.995, -25.187 | DBUA:BRY 025 | OP086105 | **x** |  |
|  | MB10 | Santa Maria, Azores | 27 | 36.995, -25.187 | DBUA:BRY 029 | OP086108 | **x** |  |
|  | MB12 | Santa Maria, Azores | 27 | 36.995, -25.187 | DBUA:BRY 031 | OP086109 | **x** |  |
|  | GA8 | Santa Maria, Azores | 11-13 | 36.982, -25.039 | DBUA:BRY 031 | OP086110 | **x** |  |
|  | GA16 | Santa Maria, Azores | 11-13 | 36.982, -25.039 | DBUA:BRY 055 | OP086111 | *f* |  |
|  | GA17 | Santa Maria, Azores | 11-13 | 36.982, -25.039 | DBUA:BRY 057 | OP086112 | *f* |  |
|  | GA20 | Santa Maria, Azores | 11-13 | 36.982, -25.039 | DBUA:BRY 059 | OP086113 | **x** |  |
|  | GA21 | Santa Maria, Azores | 11-13 | 36.982, -25.039 | DBUA:BRY 060 | OP086114 | **x** |  |
|  | SM15-1-1 | Santa Maria, Azores | 10 | 36.982, -25.039 | Personal:BBerning | OP086119 | n.a. |  |
|  | SM15-6-1 | Santa Maria, Azores | 10 | 36.944, -25.007 | Personal:BBerning | OP086120 | n.a. |  |
|  | SM15-6-2 | Santa Maria, Azores | 10 | 36.944, -25.007 | Personal:BBerning | OP086121 | n.a. |  |
|  | BRY15 | Flores, Azores | 151 | 39.570, -31.217 | M150-BIOD 072 | OP086135 | *f* |  |
|  | BRY16 | Flores, Azores | 151 | 39.570, -31.217 | M150-BIOD 073 | OP086099 | *f* |  |
|  | BRY18 | Flores, Azores | 151 | 39.570, -31.217 | M150-BIOD 076 | OP086136 | *f* |  |
|  | BRY19 | Flores, Azores | 151 | 39.570, -31.217 | M150-BIOD 077 | OP086137 | *f* |  |
|  | BRY20 | Flores, Azores | 151 | 39.570, -31.217 | M150-BIOD 078 | OP086138 | *f* |  |
|  | BRY21 | Flores, Azores | 151 | 39.570, -31.217 | M150-BIOD 079 | OP086139 | *f* |  |
|  | BRY22 | Flores, Azores | 151 | 39.570, -31.217 | M150-BIOD 080 | OP086140 | *f* |  |
|  | BRY23 | Flores, Azores | 151 | 39.570, -31.217 | M150-BIOD 081 | OP086141 | *f* |  |
|  | BRY24 | Flores, Azores | 151 | 39.570, -31.217 | M150-BIOD 082 | OP086142 | *f* |  |
|  | BRY25 | Flores, Azores | 151 | 39.570, -31.217 | M150-BIOD 083 | OP086143 | **x** |  |
|  | BRY 100 | Terceira, Azores | 160 | 38.811, -27.335 | M150-BIOD 434 | OP086126 | *f* |  |
|  | BRY 102 | Terceira, Azores | 160 | 38.811, -27.335 | M150-BIOD 436 | OP086127 | **x** |  |
|  | BRY 104 | Terceira, Azores | 160 | 38.811, -27.335 | M150-BIOD 438 | OP086128 | *f* |  |
|  | BRY 105 | Terceira, Azores | 160 | 38.811, -27.335 | M150-BIOD 439 | OP086129 | *f* |  |
|  | BRY 130 | Terceira, Azores | 152 | 38.802, -27.071 | M150-BIOD 532 | OP086130 | **x** |  |
|  | BRY 134 | Terceira, Azores | 152 | 38.802, -27.071 | M150-BIOD 536 | OP086131 | **x** |  |
|  | BRY 139 | Terceira, Azores | 152 | 38.802, -27.07 | M150-BIOD 543 | OP086132 | **x** |  |
|  | BRY-95-1 | Terceira, Azores | 151 | 38.811, -27.335 | M150-BIOD 504 | OP086122 | n.a. |  |
|  | BRY-95-2 | Terceira, Azores | 151 | 38.811, -27.335 | M150-BIOD 504 | OP086125 | n.a. |  |
|  | SP-60m-2 | Faial, Azores | 60 | 38.520, -28.605 | Personal:BBerning-SP | - | n.a. |  |
|  | SP-60m-3 | Faial, Azores | 60 | 38.520, -28.605 | Personal:BBerning-SP | OP086145 | n.a. |  |
|  | SP-60m-5 | Faial, Azores | 60 | 38.520, -28.605 | Personal:BBerning-SP | OP086150 | n.a. |  |
|  | SP-60m-6 | Faial, Azores | 60 | 38.520, -28.605 | Personal:BBerning-SP | OP086146 | n.a. |  |
|  | SP-60m-7 | Faial, Azores | 60 | 38.520, -28.605 | Personal:BBerning-SP | OP086147 | n.a. |  |
|  | SP-60m-8 | Faial, Azores | 60 | 38.520, -28.605 | Personal:BBerning-SP | OP086144 | n.a. |  |
|  | SP-150m-1 | Faial, Azores | 150 | 38.509, -28.610 | Personal:BBerning-SP | OP086151 | n.a. |  |
|  | SP-150m-3 | Faial, Azores | 150 | 38.509, -28.610 | Personal:BBerning-SP | - | n.a. |  |
|  | SP-150m-6 | Faial, Azores | 150 | 38.509, -28.610 | Personal:BBerning-SP | OP086152 | n.a. |  |
|  | DR5.B9 | Sabrina, Azores | 150 | 37.869, -25.902 | DBUA:SAB2011 | OP086101 | **x** |  |
|  | A02 | Princess Alice Bank, Azores | 265 | 37.753, -28.991 | DOP-4193 | OP086102 | *f* |  |
|  | A08 | São Miguel, Azores | 117 | 37.77, -25.116 | DOP-5300 | OP086100 | *f* |  |
|  | A10 | Chino Bank, Azores | 371 | 37.987, -29.53 | DOP-5892 | OP086123 | **x** |  |
|  | A11 | Princess Alice Bank, Azores | 256 | 37.694, -28.935 | DOP-6132 | OP086103 | **x** |  |
|  | A12 | Princess Alice Bank, Azores | 393 | 37.990, -29.565 | DOP-6622 | OP086149 | **x** |  |
|  | A13 | Princess Alice Bank, Azores | 368 | 37.975, -29.531 | DOP-6627 | - | *f* |  |
|  | A19 | Terceira, Azores | 368 | 37.975, -29.531 | DOP-5325 | OP086124 | *f* |  |
|  | A20 | Terceira, Azores | 86 | 38.815, -27.302 | DOP-5325 | OP086134 | *f* |  |
|  | 9860B | Condor Bank, Azores | 86 | 38.815, -27.302 | DOP-9860 | OP086133 | *f* |  |
| ***R. tristis*** | BRY 127 | Terceira, Azores | 152 | 38.802, -27.071 | M150-BIOD 526 | OP086183 | *f* |  |
|  | BRY 128 | Terceira, Azores | 152 | 38.802, -27.071 | M150-BIOD 528 | OP086184 | *f* |  |
|  | BRY 129 | Terceira, Azores | 152 | 38.802, -27.071 | M150-BIOD 530 | OP086185 | **x** |  |
|  | BRY 143 | Terceira, Azores | 153 | 38.802, -27.071 | M150-BIOD 549 | OP086186 | *f* |  |
|  | BRY 195 | Santa Maria, Azores | 151 | 37.055, -25.154 | M150-BIOD 941 | OP086191 | *f* |  |
|  | BRY 196 | Santa Maria, Azores | 151 | 37.055, -25.154 | M150-BIOD 943 | OP086192 | *f* |  |
|  | BRY 198 | Santa Maria, Azores | 152 | 37.055, -25.154 | M150-BIOD 946 | OP086193 | *f* |  |
|  | BRY 204 | Santa Maria, Azores | 152 | 37.055, -25.154 | M150-BIOD 952 | OP086194 | *f* |  |
|  | BRY 216 | Santa Maria, Azores | 152 | 37.055, -25.154 | M150-BIOD 964 | OP086195 | *f* |  |
|  | BRY 220 | Santa Maria, Azores | 153 | 37.055, -25.154 | M150-BIOD 971 | OP086196 | *f* |  |
|  | BRY 222 | Santa Maria, Azores | 153 | 37.055, -25.154 | M150-BIOD 974 | OP086197 | *f* |  |
|  | BRY 224 | Santa Maria, Azores | 152 | 37.055, -25.154 | M150-BIOD 978 | OP086198 | *f* |  |
|  | BRY 225 | Santa Maria, Azores | 152 | 37.055, -25.154 | M150-BIOD 980 | OP086189 | **x** |  |
|  | BRY 226 | Santa Maria, Azores | 152 | 37.055, -25.154 | M150-BIOD 982 | OP086190 | **x** |  |
|  | DR10.B7 | Sabrina, Azores | 150 | 37.882, -25.907 | DBUA:SAB2011 | OP086188 | *f* |  |
|  | M151-50341 | São Miguel, Azores | 200 | 37.675, -25.717 | SaM-ID-50341 | OP086187 | n.a. |  |
| ***R. oceanica*** | 9869A | Condor Bank | 455 | 38.555, -29.044 | DOP-9869A | OP086154 | **x** |  |
|  | 9869B | Condor Bank | 455 | 38.555, -29.044 | DOP-9869B | OP086155 | **x** |  |
| ***Reteporella* sp. 6** | BRY 103 | Terceira, Azores | 160 | 38.811, -27.335 | M150-BIOD 437 | OP086159 | **x** |  |
|  | BRY 117 | Terceira, Azores | 277 | 38.802, -27.059 | M150-BIOD 515 | OP086162 | **x** |  |
|  | BRY 136 | Terceira, Azores | 153 | 38.803, -27.071 | M150-BIOD 539 | OP086158 | **x** |  |
|  | BRY 141 | Terceira, Azores | 152 | 38.802, -27.071 | M150-BIOD 546 | OP086161 | **x** |  |
|  | BRY 146 | Terceira, Azores | 153 | 38.802, -27.071 | M150-BIOD 553 | OP086160 | **x** |  |
|  | 10302E | Gigante Bank, Azores | 493 | 38.975, -29.852 | DOP-10302 | OP086156 | **x** |  |
|  | 10303 | Gigante Bank, Azores | 402 | 39.008, -29.929 | DOP-10303 | OP086157 | **x** |  |
| ***Reteporella* sp. 7** | BRY 159 | Santa Maria, Azores | 152 | 38.802, -27.071 | M150-BIOD 720 | OP086175 | *f* |  |
|  | BRY 170 | Santa Maria, Azores | 144 | 37.047, -25.057 | M150-BIOD 719 | OP086176 | **x** |  |
|  | BRY 217 | Santa Maria, Azores | 152 | 37.047, -25.057 | M150-BIOD 966 | OP086177 | **x** |  |
|  | BRY 218 | Santa Maria, Azores | 153 | 37.055, -25.154 | M150-BIOD 968 | OP086178 | **x** |  |
|  | BRY 223 | Santa Maria, Azores | 153 | 37.055, -25.154 | M150-BIOD 976 | OP086181 | **x** |  |
|  | BRY-211-4 | Santa Maria, Azores | 153 | 37.055, -25.154 | M150-BIOD 959 | OP086174 | n.a. |  |
|  | BRY-221-2 | Santa Maria, Azores | 152 | 37.055, -25.154 | M150-BIOD 972 | OP086173 | n.a. |  |
|  | M151-50332 | São Miguel, Azores | 153 | 37.055, -25.154 | SaM-ID-50332 | OP086167 | n.a. |  |
|  | M151-100 113 | São Miguel, Azores | 544 | 37.670, -25.713 | SaM-ID-100 113 | OP086166 | n.a. |  |
|  | DR2.B2 A | Sabrina, Azores | 309 | 37.675, -25.717 | DBUA:SAB2011 | OP086170 | **x** |  |
|  | DR2.B2 B | Sabrina, Azores | 50-100 | 37.850, -25.667 | DBUA:SAB2011 | OP086171 | *f* |  |
|  | DR2.B2 C | Sabrina, Azores | 50-100 | 37.850, -25.667 | DBUA:SAB2011 | OP086172 | **x** |  |
|  | DR3.B13 A | Sabrina, Azores | 50-100 | 37.850, -25.667 | DBUA:SAB2011 | OP086168 | *f* |  |
|  | DR3.B13 B | Sabrina, Azores | 200 | 37.855, -25.683 | DBUA:SAB2011 | OP086169 | *f* |  |
|  | DR4.B13 | Sabrina, Azores | 200 | 37.855, -25.683 | DBUA:SAB2011 | OP086179 | *f* |  |
|  | A07 | Santa Maria, Azores | 140 | 37.873, -25.900 | DOP-5282 | OP086180 | **x** |  |
|  | 10302A | Gigante Bank, Azores | 138 | 36.911, -25.022 | DOP-10302 | OP086164 | *f* |  |
|  | 10302C | Gigante Bank, Azores | 493 | 38.975, -29.852 | DOP-10302 | OP086165 | **x** |  |
|  | BRY 257 | Formigas, Azores | 150 | 37.274, -24.755 | M150-BIOD 1140 | OP086163 | **x** |  |

**Table S2.** Complete list of SSR primers designed in this study, listing their sequences, reference libraries, repetition motifs, and number of repeats in the original sequence from which the primers were designed. The mixes in which primers were included for the multiplex PCRs are also indicated. The last six primers failed during single PCR tests and were omitted from any SSR genotyping. It is also indicated if the primer was informative for population genetic analyses or if it was excluded from the dataset after applying criteria for missing data (> 50%). The number of alleles and length range of the loci analysed are indicated in the last two columns.

| Primer/ Loci | Forward (5’-3’) | Reverse (5’-3’) | Reference library | Repetition motif | Mix | Population genetic analyses? | # alleles | Loci length range |
| --- | --- | --- | --- | --- | --- | --- | --- | --- |
| Bry1_CATA | GGTTGGTTGATTGTGACCAG | GTAGGGAGCTAGCCAATTGA | BRY16 (*R. atlantica*) | (CATA)6 | 1 | Yes | 25 | 394-407 bp |
| Bry2_TATG | TACACCCAAATTTCCTGCCA | GTGCCTCCCACAAATTCATG | BRY16 (*R. atlantica*) | (TATG)5 | 3 | Yes | 15 | 403-411 bp |
| Bry3_TATC | GTTTGGAGCTCAACATTCGT | ACGCCATCATCACTTACTGT | BRY141 (*Reteporella* sp. 6) | (TATC)23 | 3 | Yes | 31 | 252-343 bp |
| Bry4_GGTA | TCTGCAGAGCCGAATCATTA | CCGGGACTATGTGAAGCTAT | BRY141 (*Reteporella* sp. 6) | (GGTA)5 | 4 | Yes | 35 | 331-353 bp |
| Bry6_TACC | TATGACAGTTCCGATGGACG | CATCCTAAAGGCACAACAGC | BRY141 (*Reteporella* sp. 6) | (TACC)6 | 4 | Yes | 39 | 312-370 bp |
| Bry7_GATA | GCCAAGGAGTCCTACATGTC | ATTCTGGAAACGAACTGGGA | BRY141 (*Reteporella* sp. 6) | (GATA)6 | 4 | Yes | 40 | 258-362 bp |
| Bry8_TGTC | CCCTACCCTTGTCATACACT | TGGTAAGGCTATTGTTGACCA | BRY141 (*Reteporella* sp. 6) | (TGTC)11 | 4 | Yes | 66 | 253-316 bp |
| Bry9_AATA | ACCATGTTCCTCACTTTGGT | TAGTCCGCAAATGACCAGA | BRY141 (*Reteporella* sp. 6) | (AATA)5 | 2 | Yes | 29 | 398-428 bp |
| Bry10_CACTA | GTCCAAAGTTGATCACTGCG | TCGCCACTGCTCTATTTGAT | BRY141 (*Reteporella* sp. 6) | (CACTA)5 | 1 | Yes | 46 | 291-361 bp |
| Bry11_CCTA | GGATAGCTTCTTGGCTTGGA | GTCCCACAAGCTCACATTTG | BRY141 (*Reteporella* sp. 6) | (CCTA)5 | 2 | Yes | 42 | 253-348 bp |
| Bry12_CTGT | TCAACCGCTATAGCCATTGT | TGTGTGTTCTTGCAGACTCT | BRY141 (*Reteporella* sp. 6) | (CTGT)6 | 1 | Yes | 16 | 302-323 bp |
| Bry14_ATAC | TGTCTCGAAGCTGTCTTGAA | AACATGGCTAACTCACCCTC | BRY141 (*Reteporella* sp. 6) | (ATAC)6 | 3 | Yes | 39 | 341-404 bp |
| Bry15_CATA | TCCATCCATACGTGTATGCA | AGTGCAAACTCCCACAATTG | BRY141 (*Reteporella* sp. 6) | (CATA)5 | 2 | Yes | 28 | 299-330 bp |
| Bry16_CCTA | TTAAGTCACTGCAAGTTGGC | CACAACTGTAGTGGTGTGTAA | BRY141 (*Reteporella* sp. 6) | (CCTA)8 | 3 | Yes | 38 | 275-374 bp |
| Bry18_ATAC | ACATCGAATTTCAGGTTGCT | CGTGTACAACAAAGCACAGA | BRY141 (*Reteporella* sp. 6) | (ATAC)5 | 2 | No; > 50% missing | - | 344-384 bp |
| Bry19_TGTC | CTGATGTAGCGTTGTAGAGC | CCACAACAAATTGAGCCCAT | BRY141 (*Reteporella* sp. 6) | (TGTC)6 | 2 | Yes | 52 | 295-332 bp |
| Bry21_GTTA | TGCTTTCCTAGTGCTCATGT | TGCGGAACTCCACATGTTAT | BRY141 (*Reteporella* sp. 6) | (GTTA)8 | 1 | Yes | 32 | 250-345 bp |
| Bry22_TGTC | GCGGTTATTCACCCAGACT | AAGGCCACAAAAGCTGAAAG | BRY141 (*Reteporella* sp. 6) | (TGTC)5 | 3 | Yes | 31 | 323-348 bp |
| Bry23_CATA | ACAACTCCTCATTCCAGACC | AAACTCCAGAAGTGGCTGAA | BRY141 (*Reteporella* sp. 6) | (CATA)6 | 4 | Yes | 21 | 284-309 bp |
| Bry24_CAA | TCAGGCAATACGAGCAGAAT | ACACAGGAGCTGGTACCTAG | BRY141 (*Reteporella* sp. 6) | (CAA)10 | 2 | Yes | 39 | 390-463 bp |
| Bry25_TGT | CAGAAAAGGTTGATGCGGTC | GGCTTCCTTTGAGGGATTTC | BRY141 (*Reteporella* sp. 6) | (TGT)9 | 4 | No; > 50% missing |  | 323-353 bp |
| Bry26_ATA | GTTGTGACAGATCGTGACAG | GTGAGTGTGACAGCCATAGA | BRY141 (*Reteporella* sp. 6) | (ATA)9 | 2 | Yes | 15 | 314-386 bp |
| Bry27_ATA | GGTTTACAACCGGCTTTACC | TCCATAGTGGTGCTTGTTCA | BRY141 (*Reteporella* sp. 6) | (ATA)9 | 4 | No; > 50% missing |  | 324-348 bp |
| Bry28_TTA | ACCTACACTGACAGCAACAA | ATTACTTGATGACGGGTCCC | BRY141 (*Reteporella* sp. 6) | (TTA)8 | 1 | Yes | 28 | 295-345 bp |
| Bry30_TTA | CTCTCGTGTGTCCTGGAATT | GCTATTGTACACTTGCTAGCC | BRY141 (*Reteporella* sp. 6) | (TTA)8 | 2 | Yes | 42 | 280-324 bp |
| Bry31_TAA | ATCCTCGACTCCGTCATTTT | GCAAGTTGCATTCACAGAGT | BRY141 (*Reteporella* sp. 6) | (TAA)9 | 3 | Yes | 25 | 292-314 bp |
| Bry32_TTG | TCTGTTGTTGAGGGGTTTGT | CAATGGCTTCATTACCGCAT | BRY141 (*Reteporella* sp. 6) | (TTG)13 | 3 | Yes | 46 | 281-329 bp |
| Bry33_ACT | CATACACTCACTGCTTCCCT | GACTGCATTGGTCAGGACTA | BRY141 (*Reteporella* sp. 6) | (ACT)8 | 1 | Yes | 38 | 284-306 bp |
| Bry34_AAT | GACTTCAACACAGGGTAAGAA | GTACGGCTACTTTGTGCAAG | BRY141 (*Reteporella* sp. 6) | (AAT)8 | 4 | No; > 50% missing |  | 285-304 bp |
| Bry36_TAA | TCACGCATTAGCCTACACAT | CTAGACAGCACTGGCATGTA | BRY141 (*Reteporella* sp. 6) | (TAA)8 | 2 | Yes | 41 | 282-301 bp |
| Bry37_TAT | TGTCCAACACTTACACCTTCA | TTTTCAGACACTGCTCAAGC | BRY141 (*Reteporella* sp. 6) | (TAT)8 | 1 | Yes | 37 | 283-312 bp |
| Bry38_TTA | GCTGGGTGTCTTGCATTATG | CTAGTCCCTAATTGTGCAACG | BRY141 (*Reteporella* sp. 6) | (TTA)17 | 3 | Yes | 35 | 265-309 bp |
| Bry39_TAC | ATAATCTCCTCTACTCACCCC | ATACCATACGTACTGGTGGC | BRY141 (*Reteporella* sp. 6) | (TAC)15 | 1 | Yes | 39 | 267-321 bp |
| Bry40_ACT | CGCAATGGCTAAGGCAAATT | GAGCCACTACTACAGCAGAT | BRY141 (*Reteporella* sp. 6) | (ACT)9 | 3 | No; > 50% missing |  | 288-303 bp |
| Bry41_ACA | AGGCGAGTACACAAATGTAAC | ACGTGGAGATAGAGGATTTGG | BRY141 (*Reteporella* sp. 6) | (ACA)8 | 1 | Yes | 58 | 273-312 bp |
| Bry42_AATA | ATAGCACTCTGTACTCGCAT | TTGCGCAACTTCCAGATTTC | BRY141 (*Reteporella* sp. 6) | (AATA)5 | 4 | Yes | 38 | 285-316 bp |
|  |  |  |  |  |  |  |  |  |
| Failed during single PCR test | | | | | | |  |  |
| Bry5_ATAC | CAGGAGATGCAGAGTAAGCA | TCTCACCCACAAATGCACTA | BRY141 (*Reteporella* sp. 6) | (ATAC)5 | - | No |  |  |
| Bry13_CACCA | TCTGGTACCGGTACAATCAA | TAGGTGGCAATGCATCTCTT | BRY141 (*Reteporella* sp. 6) | (CACCA)5 | - | No |  |  |
| Bry17_TATG | GCATGTATGTGCATGATGATG | AATTGCTGCCACAAGAATGG | BRY141 (*Reteporella* sp. 6) | (TATG)15 | - | No |  |  |
| Bry20_ATGT | TGCACATAGAAAAGGGTCGA | GGTCGCCTGTCAACAAATAC | BRY141 (*Reteporella* sp. 6) | (ATGT)6 | - | No |  |  |
| Bry29_AAC | GGTGATGATTGGGTAGTGGA | TCAGCTACTGTAGTGAAGTGT | BRY141 (*Reteporella* sp. 6) | (AAC)20 | - | No |  |  |
| Bry35_AAT | GACTTCAACACAGGGTAAGAA | GTACGGCTACTTTGTGCAAG | BRY141 (*Reteporella* sp. 6) | (AAT)8 | - | No |  |  |

**Table S3****.** Estimates of genetic diversity per species based on: **a)** the mitochondrial marker COI; **b)** the SSR-GBAS dataset. Estimates of the number of haplotypes (h), haplotype (Hd ± standard deviation) and nucleotide (π) diversity in the COI dataset, conducted in DnaSP v6. Estimates of the mean number of alleles (Na), observed (Ho) and expected (He) heterozygosity in the SSR-GBAS dataset conducted in GenAlEx v6.51. Number of individuals (n) assigned to each group is depicted within brackets; the number of localities sampled for each species (per dataset) is shown in the second column (# locs). Genetic diversity indices in R. atlantica are estimated for all representatives, as well as separately for deep and shallow lineages.

| **a)** |  | **# locs** | **h** | **Hd** | **π** |
| --- | --- | --- | --- | --- | --- |
|  | ***R. atlantica* – all** (n=53) | 8 | 16 | 0.858 ± 0.029 | 0.01724 |
|  | *R. atlantica* – deep (n=35) | 7 | 13 | 0.850 ± 0.040 | 0.01410 |
|  | *R. atlantica* – shallow (n=18) | 1 | 3 | 0.307 ± 0.132 | 0.00058 |
|  | ***Reteporella* sp. 6** (n=7) | 2 | 4 | 0.810 ± 0.130 | 0.01104 |
|  | ***Reteporella* sp. 7** (n=18) | 3 | 7 | 0.824 ± 0.061 | 0.00848 |
|  | ***R. tristis*** (n=16) | 3 | 4 | 0.700 ± 0.090 | 0.00900 |
|  | **All** (n=95) | 9 | 31 | 0.939 ± 0.011 | 0.08799 |
|  |  |  |  |  |  |
| **b)** |  | **# locs** | **Na** | ***Ho*** | ***He*** |
|  | ***R. atlantica*** (n=20) | 7 | 16.452 | 0.477 | 0.859 |
|  | *R. atlantica* – deep (n=9) | 4 | 10.129 | 0.546 | 0.845 |
|  | *R. atlantica* – shallow (n=11) | 3 | 6.774 | 0.408 | 0.658 |
|  | ***Reteporella* sp. 6** (n=7) | 2 | 6.935 | 0.528 | 0.794 |
|  | ***Reteporella* sp. 7** (n=8) | 3 | 7.645 | 0.573 | 0.783 |
|  | ***R. tristis*** (n=3) * | 2 |  |  |  |
|  | ***R. oceanica*** (n=2) * | 1 |  |  |  |
|  | *Estimates not applicable to species with less than 5 individuals. | | | | |

**Table S4.** Hardy–Weinberg Equilibrium (HWE) tests and frequency of null allele estimates per species, conducted on the SSR-GBAS dataset using GenAlEx v6.5 and FreeNA, respectively. Significant (* p < 0.05, ** p < 0.01, *** p < 0.001) deviations from HWE are highlighted in bold. Monomorphic loci (mono), frequency of null alleles, and impossibility to estimate the parameter (-) are also depicted. For R. atlantica, these parameters were are estimated for all representatives, as well as separately for deep and shallow lineages.

| **Primer** | **R. tristis** | | **R. oceanica** | | **Reteporella sp. 6** | | **Reteporella sp. 7** | | **R. atlantica – all** | | **R. atlantica** – deep | | **R. atlantica** – shallow | | |
| --- | --- | --- | --- | --- | --- | --- | --- | --- | --- | --- | --- | --- | --- | --- | --- |
|  | **HWE** | **f(null)** | **HWE** | **f(null)** | **HWE** | **f(null)** | **HWE** | **f(null)** | **HWE** | **f(null)** | **HWE** | **f(null)** | **HWE** | **f(null)** |  |
| Bry1  CATA | *-* | *All null* | *mono* | 0.00100 | 0.464 | 0 | 0.317 | 0 | **0.009 **** | 0.08777 | 0.746 | 0.00035 | 0.991 | 0.00001 |  |
| Bry2  TATG | 0.386 | 0 | - | *All null* | 0.244 | 0.08587 | 0.135 | 0.01022 | **0.002 **** | 0.30986 | 0.059 | 0.28279 | 0.106 | 0.24535 |  |
| Bry3  TATC | *-* | *All null* | 0.317 | 0 | 0.285 | 0.00058 | 0.317 | 0 | **0.000 ***** | 0.32623 | **0.003 **** | 0.40385 | **0.004 **** | 0.24793 |  |
| Bry4  GGTA | 0.174 | 0 | 0.637 | 0 | 0.317 | 0.08739 | 0.227 | 0.00035 | **0.000 **** | 0.18003 | 0.092 | 0.10645 | 0.286 | 0.10059 |  |
| Bry6  TACC | 0.174 | 0 | - | *All null* | 0.299 | 0.00041 | 0.073 | 0 | **0.000 ***** | 0.13688 | **0.009 **** | 0.14934 | 0.671 | 0 |  |
| Bry7  GATA | 0.112 | 0.00058 | 0.317 | 0 | 0.073 | 0.24490 | **0.037 *** | 0.21429 | **0.000 ***** | 0.38246 | **0.001 ***** | 0.34974 | **0.000 ***** | 0.33715 |  |
| Bry8  TGTC | 0.532 | 0 | 0.157 | 0.33333 | 0.297 | 0.00045 | 0.469 | 0.00041 | 0.090 | 0.02147 | 0.336 | 0.00033 | 0.285 | 0 |  |
| Bry9  AATA | 0.386 | 0 | - | *All null* | **0.006 **** | 0.40322 | 0.626 | 0.06986 | **0.000 ***** | 0.03100 | 0.182 | 0.05855 | **0.001 **** | 0 |  |
| Bry10  CACTA | 0.392 | 0 | 0.157 | 0 | **0.039 *** | 0.24490 | 0.507 | 0.04346 | **0.000 ***** | 0.17511 | 0.063 | 0.17771 | 0.053 | 0.11949 |  |
| Bry11  CCTA | *mono* | 0.00100 | 0.261 | 0.00071 | 0.400 | 0 | **0.037*** | 0.31023 | **0.001 ***** | 0.16582 | 0.444 | 0 | **0.004 **** | 0.27992 |  |
| Bry12  CTGT | *mono* | 0.00100 | 0.637 | 0 | 0.112 | 0.00082 | **0.023 *** | 0.38361 | 0.107 | 0.03511 | 0.343 | 0.17564 | 0.591 | 0 |  |
| Bry14  ATAC | 0.729 | 0 | 0.637 | 0 | **0.008 **** | 0.38961 | 0.052 | 0.19821 | **0.000 ***** | 0.00002 | 0.318 | 0 | **0.000 ***** | 0.00002 |  |
| Bry15  CATA | 0.112 | 0.00082 | *mono* | 0.00100 | **0.012 *** | 0.19353 | **0.001 ***** | 0.38246 | **0.010 **** | 0.29836 | **0.018 *** | 0.31746 | 0.261 | 0.00071 |  |
| Bry16  CCTA | 0.083 | 0.31500 | 0.157 | 0.33333 | **0.026 *** | 0.16870 | 0.090 | 0.28481 | **0.000 ***** | 0.34864 | **0.001 ***** | 0.34974 | **0.001 **** | 0.30597 |  |
| Bry19  TGTC | 0.174 | 0 | 0.423 | 0 | 0.143 | 0.20000 | 0.416 | 0.00035 | **0.000 ***** | 0.24955 | 0.077 | 0.17582 | **0.020 *** | 0.26421 |  |
| Bry21  GTTA | 0.083 | 0 | - | *All null* | 0.092 | 0.24000 | 0.082 | 0.21827 | **0.000 ***** | 0.24927 | **0.013 *** | 0.09524 | 0.214 | 0.09524 |  |
| Bry22  TGTC | 0.174 | 0 | *mono* | 0.00100 | 0.058 | 0.18204 | 0.281 | 0.13098 | **0.000 ***** | 0.14299 | 0.227 | 0.04770 | 0.875 | 0.06392 |  |
| Bry23  CATA | 0.729 | 0 | *mono* | 0.00100 | 0.284 | 0 | **0.011 *** | 0.08333 | **0.000 ***** | 0.18221 | 0.051 | 0.06392 | *Mono* | 0.00100 |  |
| Bry24  CAA | 0.392 | 0 | 0.572 | 0 | 0.516 | 0 | 0.344 | 0.02366 | **0.014 *** | 0.08678 | 0.511 | 0 | **0.034 *** | 0.00100 |  |
| Bry26  ATA | 0.317 | 0 | - | *All null* | 0.062 | 0.42857 | **0.049 *** | 0.33017 | 0.066 | 0.27422 | 0.125 | 0.15732 | 0.158 | 0.23618 |  |
| Bry28  TTA | *mono* | 0.00100 | 0.637 | 0 | 0.100 | 0.30000 | **0.029 *** | 0.30949 | **0.000 ***** | 0.34217 | **0.005 **** | 0.28692 | **0.004 **** | 0.32770 |  |
| Bry30  TTA | 0.174 | 0 | 0.637 | 0 | 0.451 | 0 | 0.110 | 0.08333 | **0.007 **** | 0.07125 | 0.065 | 0.03996 | 0.508 | 0.03903 |  |
| Bry31  TAA | 0.317 | 0 | *mono* | 0.00100 | **0.023 *** | 0.29154 | 0.317 | 0 | **0.000 ***** | 0.21439 | **0.038 *** | 0.15558 | 0.067 | 0.14091 |  |
| Bry32  TTG | 0.112 | 0.00058 | 0.637 | 0 | 0.261 | 0.00071 | 0.335 | 0.02366 | 0.227 | 0.04445 | 0.301 | 0.00038 | 0.966 | 0.00035 |  |
| Bry33  ACT | *Mono* | 0.00100 | 0.317 | 0 | 0.126 | 0.18555 | 0.062 | 0.42857 | **0.027 *** | 0.16154 | 0.092 | 0.10645 | 0.511 | 0.14742 |  |
| Bry36  TAA | 0.112 | 0.00082 | 0.637 | 0 | **0.012 *** | 0.34958 | 0.289 | 0.12689 | **0.000 ***** | 0.30251 | 0.060 | 0.26617 | **0.040 *** | 0.27443 |  |
| Bry37  TAT | 0.532 | 0 | 0.157 | 0 | 0.451 | 0 | 0.286 | 0.08333 | 0.073 | 0.24490 | 0.247 | 0.13333 | 0.157 | 0.33333 |  |
| Bry38  TTA | 0.392 | 0 | *mono* | 0.00100 | 0.464 | 0.07528 | 0.336 | 0.00041 | **0.000 ***** | 0.34046 | **0.002 **** | 0.34562 | **0.000 ***** | 0.25508 |  |
| Bry39  TAC | 0.083 | 0.31500 | 0.317 | 0 | 0.214 | 0.09524 | 0.065 | 0.00071 | **0.000 ***** | 0.30708 | **0.005 **** | 0.30303 | 0.197 | 0.23455 |  |
| Bry41  ACA | 0.532 | 0 | 0.423 | 0 | 0.206 | 0.08782 | 0.345 | 0.00038 | **0.000 ***** | 0.20445 | 0.080 | 0.13333 | **0.005 **** | 0.22688 |  |
| Bry42  AATA | 0.112 | 0.00058 | 0.637 | 0 | 0.087 | 0.19682 | **0.018 *** | 0.31746 | **0.000 ***** | 0.29146 | **0.006 **** | 0.32604 | **0.002 ***** | 0.32604 |  |

**Table S5.** Hierarchical analysis of molecular variance based on the SSR-GBAS dataset among: **a)** species: R. atlantica, R. tristis, R. oceanica, Reteporella spp. 6 and 7; **b)** geographical localities: Gigante Bank, Condor Bank, Princess Alice Bank, Terceira, São Miguel, Santa Maria, and shallow waters around Santa Maria; **c)** geographical locations previously mentioned, excluding shallow waters. Analysis performed in GenAlEx v6.51, with 999 permutations. df = degree of freedom; SS = sum of squares; MS = mean squares; Est. Var. = estimate of variance; % = percentage of total variation.

| *a)* | Among species | | | | | |  |
| --- | --- | --- | --- | --- | --- | --- | --- |
|  | **Source** | ***df*** | ***SS*** | ***MS*** | ***Est. Var.*** | ***%*** | |
|  | Among populations | 4 | 167.463 | 41.668 | 1.659 | 11% | |
|  | Among individuals | 35 | 685.962 | 19.599 | 6.481 | 44% | |
|  | Within individuals | 45 | 265.500 | 6.638 | 6.638 | 45% | |
|  | Total | 79 | 1118.925 |  | 14.777 | 100% | |
|  |  |  |  |  |  |  | |
| *b)* | **Among geographical localities** | | | | | |  |
|  | **Source** | ***df*** | ***SS*** | ***MS*** | ***Est. Var.*** | ***%*** | |
|  | Among populations | 6 | 265.680 | 43.280 | 2.527 | 17% | |
|  | Among individuals | 31 | 547.426 | 17.659 | 5.520 | 38% | |
|  | Within individuals | 38 | 251.500 | 6.618 | 6.618 | 45% | |
|  | Total | 75 | 1058.605 |  | 14.665 | 100% | |
|  |  |  |  |  |  |  | |
| *c)* | **Among geographical localities, except Santa Maria-shallow water** | | | | | |  |
|  | **Source** | ***df*** | ***SS*** | ***MS*** | ***Est. Var.*** | ***%*** | |
|  | Among populations | 5 | 170.465 | 34.893 | 2.003 | 14% | |
|  | Among individuals | 21 | 382.517 | 18.215 | 5.515 | 38% | |
|  | Within individuals | 27 | 194.000 | 7.185 | 7.185 | 49% | |
|  | Total | 53 | 750.981 |  | 14.703 | 100% | |

**Table S6.** Hierarchical analysis of molecular variance, based on the COI dataset among: **a)** species: R. tristis, R. atlantica, R. oceanica, Reteporella spp. 6 and 7; **b)** geographical localities: Condor Bank, Gigante Bank, Princess Alice Bank, Terceira, São Miguel, Santa Maria, and shallow waters around Santa Maria. Analysis performed in GenAlEx v6.51, with 999 permutations. df = degree of freedom; SS = sum of squares; MS = mean squares; Est. Var. = estimate of variance; % = percentage of total variation.

| *a)* | Among species | | | | | |
| --- | --- | --- | --- | --- | --- | --- |
|  | **Source** | ***df*** | ***SS*** | ***MS*** | ***Est. Var.*** | ***%*** |
|  | Among populations | 4 | 1789.496 | 447.374 | 29.202 | 83% |
|  | Within populations | 92 | 568.164 | 6.176 | 6.176 | 17% |
|  | Total | 96 | 2357.660 |  | 35.378 | 100% |
|  |  |  |  |  |  |  |
| *b)* | **Among geographical localities** | | | | | |
|  | **Source** | ***df*** | ***SS*** | ***MS*** | ***Est. Var.*** | ***%*** |
|  | Among populations | 8 | 1049.615 | 131.202 | 11.355 | 43% |
|  | Within populations | 87 | 1296.156 | 14.898 | 14.898 | 57% |
|  | Total | 95 | 2345.771 |  | 26.254 | 100% |

**Figure S1. Principal coordinates analyses based on the SSR-GBAS dataset of *Reteporella* from the Azores.** Analyses partitioned by species (**A-B**) and geographical localities (**C-D**). **A)** Variance explained by axis 1 (11.4 %) and axis 2 (7.29 %) in the analysis by species; **B)** Variance explained by axis 1 (11.4 %) and axis 3 (5.96%) in the analysis by species; **C)** Variance explained by axis 1 (11.4 %) and axis 2 (7.29 %) in the analysis by locality; **D)** Variance explained by axis 1 (11.4 %) and axis 3 (5.96%) in the analysis by locality. PCoAs conducted in GenAlEx v6.5 (Peakall & Smouse, 2006, 2012); groups in study are coded by colour and icon shape.


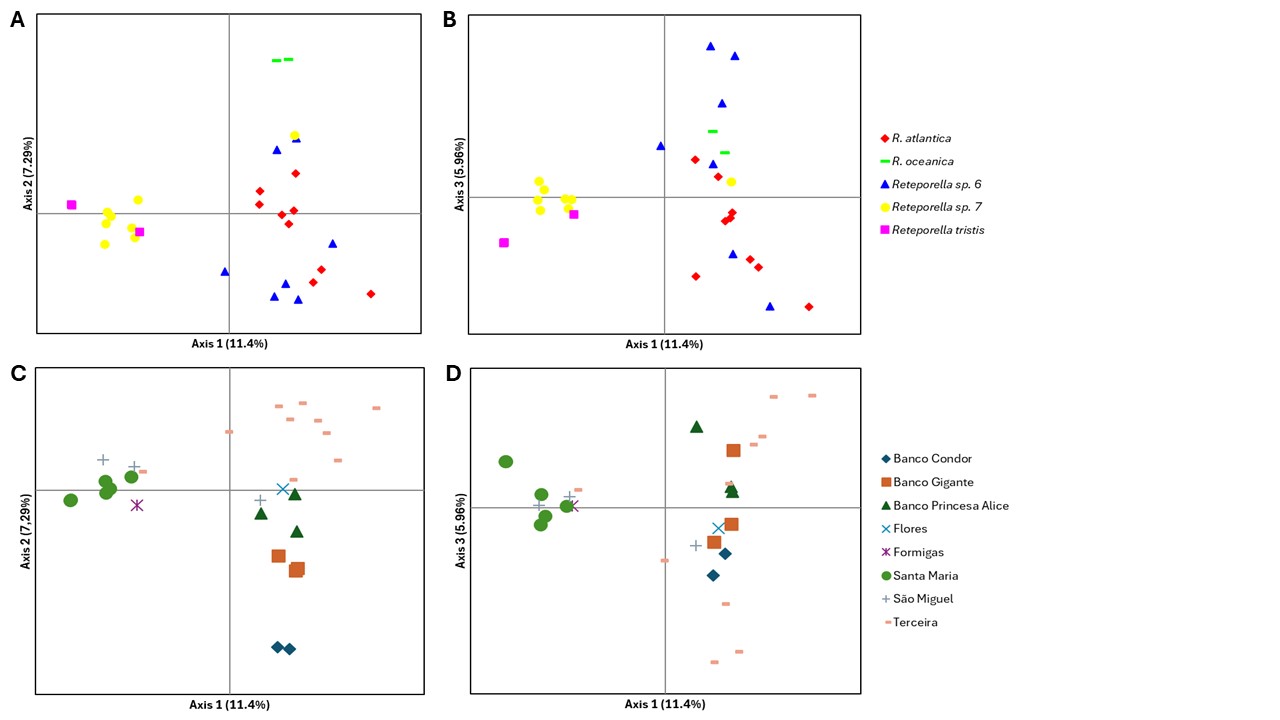


**Figure S2**. SSR-loci-specific genetic variation among groups partitioned by in species and geographical localities. **a)** Number of SSR loci for which the genetic variation among groups was estimated into the following intervals: 0-12.5 %, 12.5-25%, 25-37.5 %, 37.5-50%. **b)** SSR-loci estimates of genetic variation among groups estimated in the AMOVA. Analysis performed in GenAlEx v6.51 on the SSR-GBAS dataset, with 999 permutations.


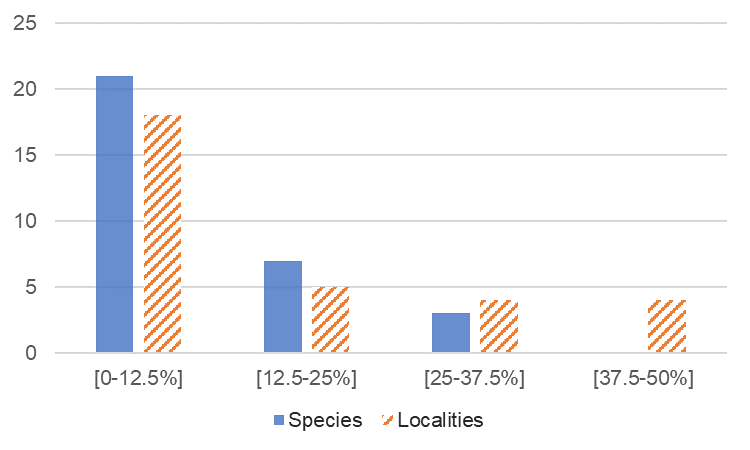

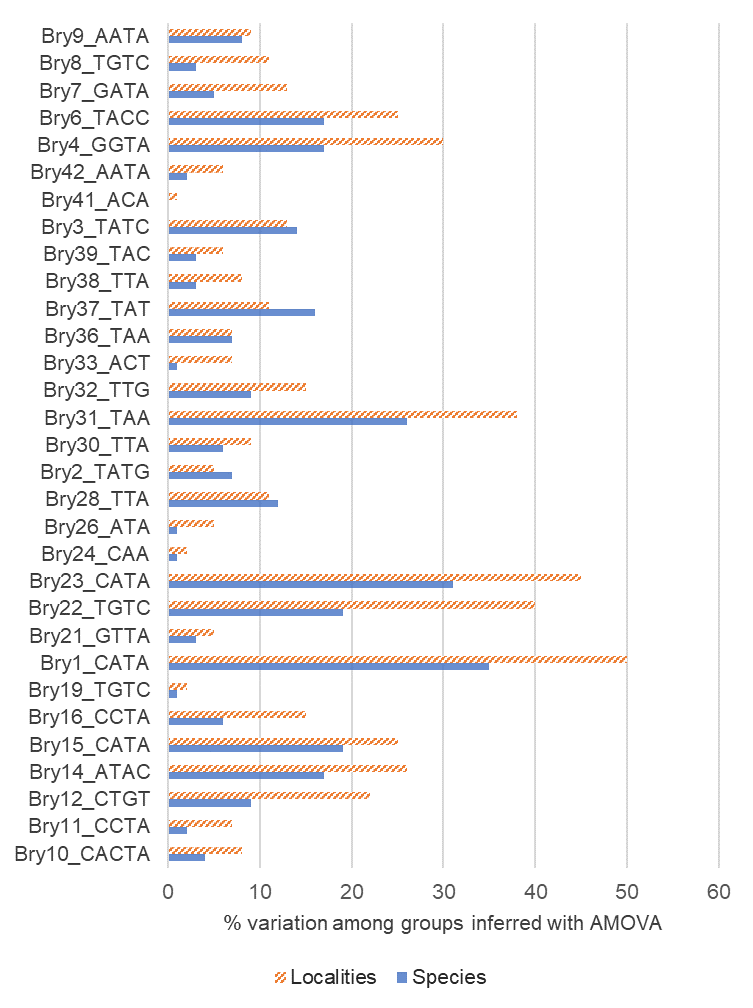


***b)***

***a)***
